# Improved Syntheses of the mGlu_5_ Antagonists MMPEP and MTEP Using Sonogashira Cross-Coupling

**DOI:** 10.3390/ph11010024

**Published:** 2018-02-20

**Authors:** Boshuai Mu, Linjing Mu, Roger Schibli, Simon M. Ametamey, Selena Milicevic Sephton

**Affiliations:** 1 Center of Radiopharmaceutical Sciences of ETH, PSI and USZ, Department of Chemistry and Applied Biosciences, Swiss Federal Institute of Technology, Vladimir-Prelog-Weg 4, 8093 Zurich, Switzerland; boshuai_mu@163.com (B.M.); linjing.mu@pharma.ethz.ch (L.M.); roger.schibli@psi.ch (R.S.); simon.ametamey@pharma.ethz.ch (S.M.A.); 2 Department of Nuclear Medicine, University Hospital Zurich, Ramistrasse 101, 8003 Zurich, Switzerland; 3 Molecular Imaging Chemistry Laboratory, Wolfson Brain Imaging Centre, Department of Clinical Neurosciences, University of Cambridge, Box 65 Cambridge Biomedical Campus, Cambridge CB2 0QQ, UK

**Keywords:** Sonogashira cross-coupling, MMPEP, MTEP, mGlu_5_ antagonist

## Abstract

The Sonogashira cross-coupling, a key step in the syntheses of the mGlu_5_ antagonists MMPEP and MTEP, provided an improved three-step method for the preparation of MMPEP in 62% overall yield. Using Spartan molecular modeling kit an explanation for the failure to employ analogues method in the synthesis of MTEP was sought. The DFT calculations indicated that meaningful isolated yields were obtained when the HOMO energy of the aryl halide was lower than the HOMO energy of the respective alkyne.

## 1. Introduction

**Figure 1:**
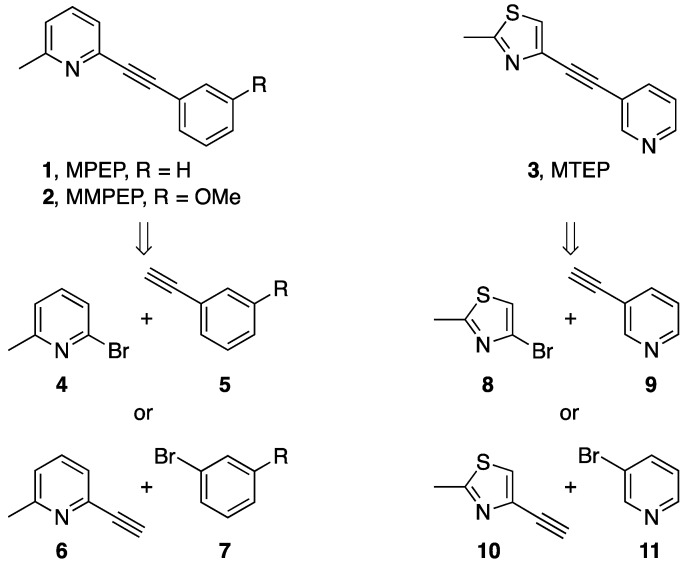
Structures of MPEP (**1**), MMPEP (**2**) and MTEP (**3**) and their assembly via the Sonogashira cross-coupling.

Metabotropic glutamate receptor subtype 5 (mGlu_5_) [1,2,3] is a 7-member transmembrane G-protein coupled receptor, which based on its pharmacology belongs to the group I of metabotropic receptors and regulates glutamate, a major neurotransmitter in the mammalian brain. Consequently, mGlu_5_ is associated with numerous central nervous system (CNS) disorders such as Parkinson’s [1,4] and Alzheimer’s [5,6] disease. For this reason mGlu_5_ has been studied extensively as a potential therapeutic target [7], resulting in the identification of 2-methyl-6-(phenylethynyl)pyridine (MPEP, **1**, Figure 1) [8,9] as a potent mGlu_5_ antagonist which presented the basis for further Structure Activity Relationship (SAR) studies. Additionally, significant efforts have been made in establishing positron emission tomography (PET) [10] radiotracers for non-invasive imaging of mGlu_5_ [11] with the aim of further assisting drug development and also as a potential diagnostic tools of CNS impairments. As a part of the on-going program on the development of fluorine-18 labelled mGlu_5_ radiotracer based on the structural scaffold of (*E*)-3-[(6-methylpyridin-2-yl)ethynyl]cyclohex-2-enone *O*-[^11^C]methyl oxime ([^11^C]-ABP688) [12,13] we recently required the two mGlu_5_ antagonists 2-((3-methoxy-phenyl)ethynyl)-6-methylpyridine (MMPEP, **2**) [14] and 2-methyl-4-(pyridine-3-ylethynyl)thiazole (MTEP, **3**, Figure 1) [15,16,17]. The key transformation in the syntheses of both mGlu_5_ antagonists was a Sonogashira cross-coupling [18].

The Sonogashira cross-coupling reaction has been used for other analogous molecules [19,20] and is characterized by the ease of synthetic manipulation required to perform the reaction, generally mild reaction conditions, including ambient temperature and relatively short reaction times, and the versatility of substrates amenable to these conditions [21]. Extensive studies on the mechanism of the Sonogashira cross-coupling allowed for postulations about the effect of electronegativity of coupling partners, where electron-donating substituents on the aryl or alkyl halide lead to a higher activation barrier and electron-withdrawing substituents on the aryl or alkyl halide lower the barrier [22]. It was hypothesized that turnover-determining step in which aryl halides participate is preceded by the end-on ligation of the halogen atom to palladium making it therefore an electron-donating step [21]. Thus, electron-donating groups with higher HOMO energy of the substrate form more stable complexes and as a consequence have higher rate determining activation barrier. On the other hand, electron-withdrawing groups with low HOMO energy, hence lower the activation barrier and facilitate oxidative insertion. Herein we report our findings about the application of the Sonogashira coupling in the syntheses of two mGlu_5_ antagonists resulting in a shorter synthetic route to MMPEP than that previously described. Rationale for the reactivity of Sonogashira partners was found through HOMO energy calculations using Density Functional Theory (DFT).

## 2. Results and Discussion

**Scheme 1:**
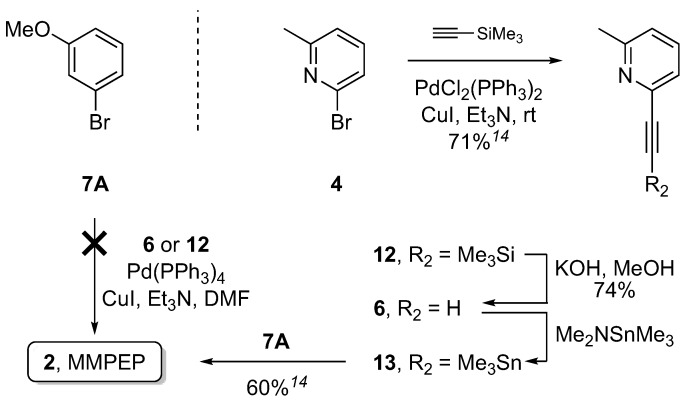
1-Bromo-3-methoxybenezene (**7A**) as reacting partner in Sonogashira coupling to access MMPEP (**2**).

The previously reported synthesis of MMPEP involved a four step route starting with commercially available bromide **4** (Scheme 1) and giving 31% overall yield [14]. In the first step, **4** was converted to alkyne **12** employing Sonogashira cross-coupling, and the trimethylsilyl functionality was then removed under basic conditions (compound **6**) and the trimethyltin group introduced onto the terminal alkyne in **13**, and **13** then reacted with aryl bromide **7A** under Stille cross-coupling reaction conditions to afford MMPEP (**2**). Due to the total number of transformations as well as the toxicity of tin, an alternative direct route to access **2** was sought. Use of the same bromide **7A** with either TMS alkyne **12** in the presence of TBAF·THF for in situ deprotection, or the deprotected equivalent **6**, formed in one step from 2-bromopyridine (see Supplementary Materials), failed to furnish MMPEP (Scheme 1). The latter had precedent in the synthesis of ABP688 [23]. In this case, 3-bromocyclohex-2-enone was coupled with **6** in 60% yield but the reaction mixture required heating to 55 °C (not shown).

**Scheme 2:**
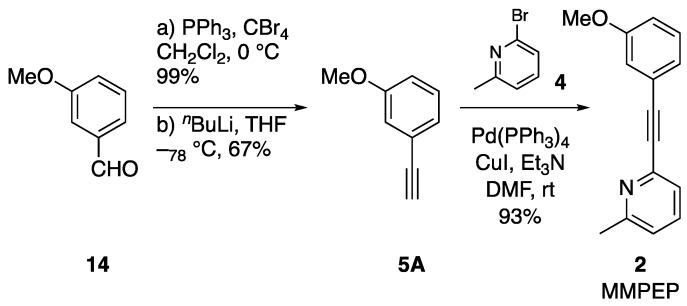
Improved three step synthesis of MMPEP (**2**) with 1-ethynyl-3-methoxybenzene (**5A**).

The failure to form the desired cross-coupling product between bromide **7A** and alkynes **12** or **6** prompted us to further explore an alternative synthetic route towards **2** (Scheme 2). For this, alkyne **5A** was obtained from 3-methoxyanisole (**14**) in two steps employing classical Corey-Fuchs reaction in 66% overall yield [15]. When alkyne **5A** was reacted directly with 2-bromo-6-methylpyridine (**4**) under Sonogashira cross-coupling conditions at ambient temperature, MMPEP (**2**) was isolated in 93% yield (Scheme 2). This demonstrated that Stille coupling was not required and that **2** can be prepared in excellent yield of 62% over only three reaction steps and on 1 g scale.

**Figure 2:**
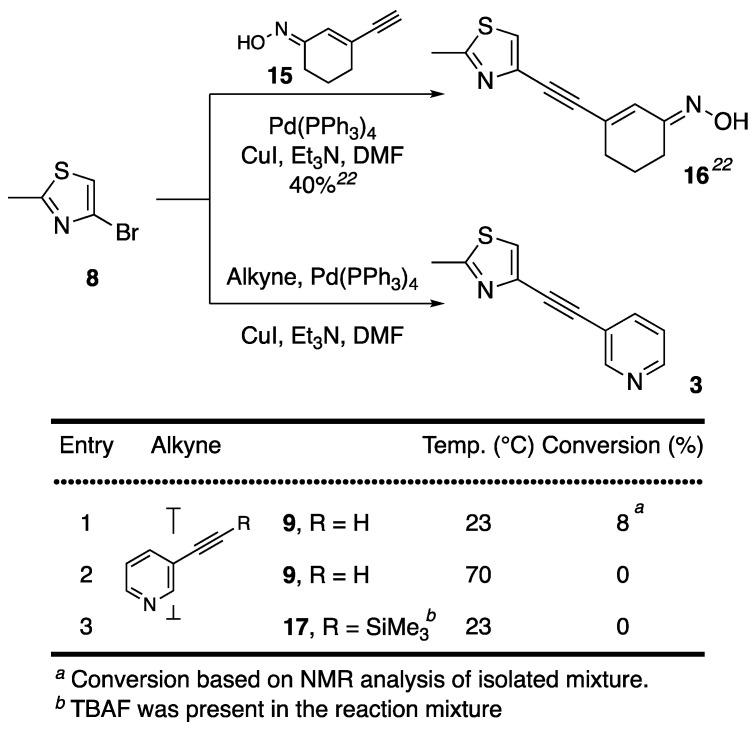
4-Bromo-2-methylthiazole (**8**) as a reacting partner in Sonogashira coupling *en route* to MTEP (**3**).

**Figure 3:**
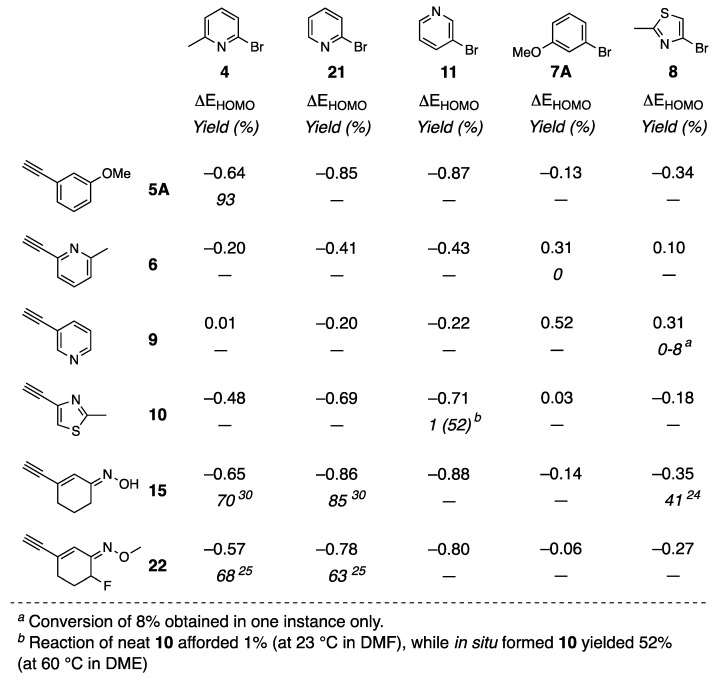
Difference in values of HOMO energies of reacting partners (ΔE_HOMO_) calculated using DFT method compared to experimental yields. Note: “—” indicates that reaction was not performed experimentally.

We next sought to explore an analogous synthetic strategy for the construction of MTEP (**3**). Our recent report on the synthesis and properties of novel ABP688 derivative (**16**, ThioABP) [24] established the successful application of Sonogashira cross-coupling between 4-bromo-2-methylthiazole (**8**) and alkyne **15** at ambient temperature in moderate 40% yield (Figure 2). The same bromide **8** in reaction with 3-ethynylpyridine (**9**), however, afforded desired product in only 8% conversion, based on the NMR analysis of isolated mixtures (Figure 2, entry 1). Analogue **3** could not be separated from the major product of the reaction, which was presumed to be result of self-coupling of alkyne **9** but remained uncharacterized. When reaction was repeated under the same conditions, it failed to yield any of the desired **3**, thus proving to be non-reproducible and an inefficient way to obtain **3**. At elevated temperature, reaction of bromide **8** with akyne **9** also failed to yield desired **3** (Figure 2, entry 2). Furthermore, the reaction was unsuccessful with silylated alkyne **17**, which was desilylated with TBAF·THF in situ (Figure 2, entry 3) therefore excluding the possibility of effects of presence of TBAF·THF in the reaction mixture on reactivity of coupling partners. The possibility of TBAF·THF effect on the success of Sonogashira cross-coupling was founded based on our previously published observations whereby in situ desilylated α-fluoroalkynes using TBAF·THF were successfully coupled to bromopyridines under analogous reaction conditions (see Figure 3, alkyne **22**) [25]. 

Our findings suggested that both reacting partners play a significant role in the cross-coupling and are determinants in the reaction success, which lead us to conduct a comparison with the previously reported synthesis of **3**.

**Scheme 3:**
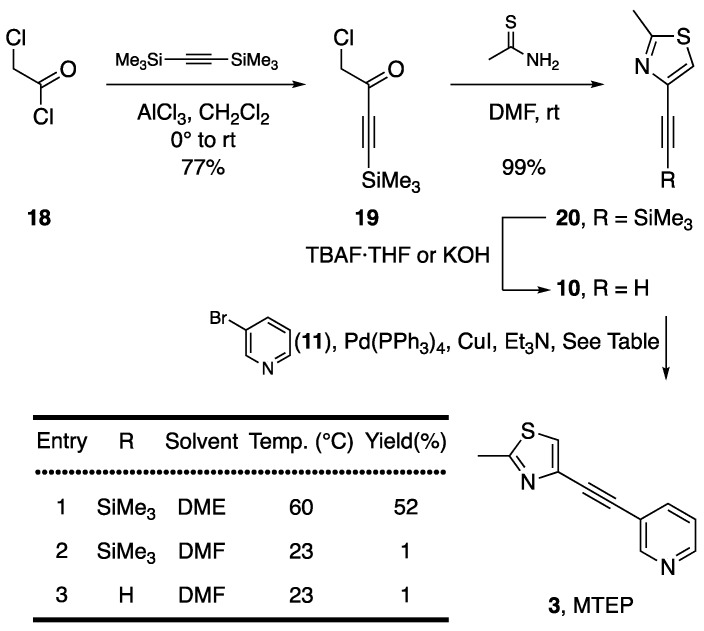
Synthesis of MTEP (**3**) at elevated reaction temperature with 4-alkynyl-2-methylthiazoles (**10** or **20**) as coupling partners.

The literature report of the synthesis of MTEP (**3**) is analogous to the one depicted in Scheme 3. Silylated alkyne **20** was prepared in two steps starting from commercially available acid chloride **18** with 76% overall yield. The Sonogashira cross-coupling between **20** and **11** was performed at 60 °C, in the presence of TBAF·THF which was added via syringe pump over several hours [15]. In our hands, this protocol afforded 52% of **3** (entry 1). 

Our synthesis thus compared favourably to the one previously published with 39% overall yield vs. 25% reported over the same number of steps. Using the same reacting partners the reaction was then performed at ambient temperature and TBAF·THF was added in one portion (entry 2, an approach previously applied successfully for the series of α-fluorinated oximes with pyridinyl bromides) [25] but this failed to give the desired coupling product in a meaningful amounts. Alkyne **20** was then desilylated to give **10** prior to its addition to the reaction mixture and attempted Sonogashira cross-coupling at ambient temperature gave only trace amounts of desired product (entry 3). To further confirm effects TBAF·THF may have **10** was prepared either as neat material when desilylation was perfomed with KOH as a base, or as a crude mixture containing TBAF·THF when desilylation was done using TBAF solution. Interestingly, either attempt afforded only trace (<1%) amounts of desired product (see Supplementary Materials). With these results in hand we aimed to assess possible reason for different reactivity of the same bromide (e.g., reaction of **8** with **15** and **9** or **17**). Considering the electronegativity of the halides as an important factor [21,22] an explanation was sought through calculation of the respective HOMO energies.

Equilibrium as well as HOMO and LUMO energies were calculated using the DFT method within the Spartan Molecular Modeling kit (the values are provided in the Supplementary Materials). The DFT method as well as the basis set used has previously been applied successfully for other computational studies on the mechanism of Sonogashira coupling [22]. The HOMO energies were calculated for both reacting partners, the halide and the alkyne. As discussed in the Introduction, it has been reported that the lower the HOMO energy of the halide, the more facile oxidative insertion of the palladium into the aryl-halide bond (i.e., more facile interaction between HOMO of the halide and the respective a1 acceptor orbital on the metal). However, our experimental data showed different reactivity of the same aryl halides, thus suggesting that HOMO energy of the aryl halide is not the only determinant of the reaction success. For this reason, HOMO energies of halides and reacting alkynes were compared with respect to the experimentally obtained yields and the difference in HOMO values between the two (ΔE_HOMO_ = E_HOMO_ (halide) − E_HOMO_ (alkyne)) is provided in Figure 3. It was found that product formation was not guaranteed if halide reacted with the alkyne which HOMO energies were of the same value or higher. Meaningful yields of desired products up to 93% were obtained when the HOMO energy of halide was lower than the HOMO energy of the reacting alkyne. In mechanistic terms, oxidative insertion of palladium to halide will proceed at the same rate for halides of equal HOMO energies, and the reaction outcome is further determined by the co-ordination of alkyne to copper(I). While the oxidative insertion of palladium (i.e., first catalytic cycle) is studied and well understood, the subsequent catalytic cycle (i.e., transmetalation) is poorly known and some studies [21] show the possibility of tandem palladium/cooper catalysis. Considering the possibility that both metals can react, and to facilitate oxidative addition, our findings suggest that the product formation is dependent on the HOMO energy of both coupling partners. Although only a subset of reacting possibilities for given halides and alkynes of interest was explored experimentally, in those cases the isolated yields were in agreement with the computationally based hypothesis. Furthermore, results in Figure 3 indicate that cyclic aliphatic alkynes outperform heterocyclic alkynes in terms of reactivity in Sonogashira coupling reactions thus showcasing importance of electronic nature of alkyne coupling partners.

**Figure 4:**
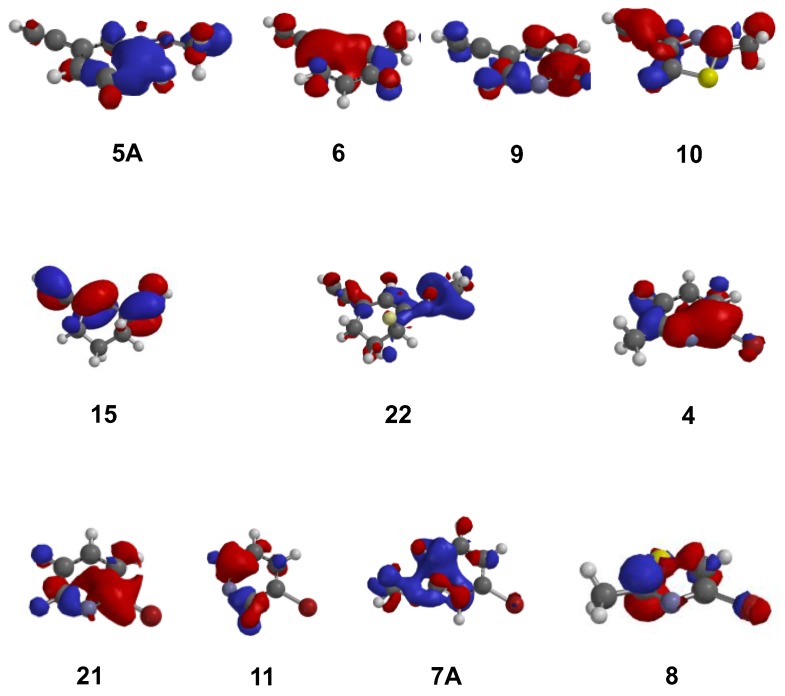
HOMO diagrams for all computationally investigated molecules (alkynes and halides) calculated using DFT method within the Spartan Molecular Modeling kit.

Furthermore, for the explored halides and alkynes HOMOs were different not only in terms of their respective energies, but their nature too. For example for alkynes **5A**, **6** and **9** HOMOs were centered on the aromatic ring unlike **10** in which HOMO was centered on the alkyne. While HOMO of oxime **15** was distributed across the conjugated system of alkyne, cyclic alkene and *N*-oxime (see Supplementary Materials), oxime **22** had HOMO centered on the *N*-oxime functionality only (Figure 4).

Similarly, for two of the bromides (**4** and **21**) HOMO was centered on the carbon bearing bromide, whereas HOMO was centered in the position *para* to the bromide-bearing carbon for bromides **11**, **7A** and **8**, thus suggesting that the nature of HOMO varied significantly amongst the investigated alkynes/halides. For this reason, HOMO energy alone may not be sufficient indicator of the complex nature of reactive species in the Sonogashira cross-coupling reaction; however our computational findings have allowed explanation for the experimental results observed in the syntheses of MMPEP and MTEP.

## 3. Materials and Methods

### 3.1. General Techniques

All reactions requiring anhydrous conditions were conducted in flame-dried glass apparatus under an atmosphere of inert gas. All chemicals and anhydrous solvents were purchased from Aldrich (St. Gallen, Switzerland) or ABCR (Karlsruhe, Germany) and used as received, unless otherwise noted. Reported density values are for ambient temperature. Preparative chromatographic separations were performed on Aldrich Science silica gel 60 (35–75 μm) and reactions followed by TLC analysis using Sigma-Aldrich (St. Gallen, Switzerland) silica gel 60 plates (2–25 μm) with fluorescent indicator (254 nm) and visualized with UV or potassium permanganate. ^1^H- and ^13^C-NMR spectra were recorded in Fourier transform mode at the field strength specified on Avance FT-NMR spectrometers (Bruker, Faellanden, Switzerland). Spectra were obtained from the specified deuterated solvents in 5 mm diameter tubes. Chemical shift in ppm is quoted relative to residual solvent signals calibrated as follows: **CDCl_3_** δ_H_ (C*H*Cl_3_) = 7.26 ppm, δ_C_ = 77.2 ppm. Multiplicities in the ^1^H-NMR spectra are described as: s = singlet, d = doublet, t = triplet, q = quartet, quint. = quintet, m = multiplet, b = broad; coupling constants are reported in Hz.

### 3.2. Syntheses

*1-Ethynyl-3-methoxybenzene* (**5A**). A one-necked round bottom flask was charged with triphenylphosphine (10.5 g, 40 mmol, 4 eq.), then carbon tetrabromide (6.63 g, 20 mmol, 2 eq.) and the yellow solid mixture was carefully dissolved in anhydrous dichloromethane (36 mL; CAUTION: vigorous reaction!) and the resulting orange mixture was allowed to cool to 0 °C (the ice bath). The heterogeneous and red in colour mixture was allowed to stir and then treated with *m*-anisaldehyde (1.2 mL, 1.36 g, 10 mmol, 1 eq., d = 1.119) dropwise over 1 min and the resulting dark orange mixture was allowed to stir at 0 °C for 30 min and then the cooling bath was removed and stirring continued at ambient temperature for 38 min. After this time the crude mixture was quenched with ice cold H_2_O (40 mL) and diluted with hexanes (25 mL) and the two layers were well shaken and separated. The aqueous phase was extracted with hexanes (5 × 25 mL). The combined organic extracts were concentrated in vacuo and the crude mixture was purified by chromatography on a silica gel column (eluting with 100% hexanes) to afford 1-(2,2-dibromovinyl)-3-methoxybenzene (2.88 g, 9.9 mmol, 99%): ^1^H-NMR (400 MHz, CDCl_3_) δ 7.46 (bs, 1H), 7.29 (t, *J* = 8.0 Hz, 1H), 7.12 (tm, *J* = 1.9 Hz, 1H), 7.09 (ddt, *J* = 7.7, 1.4, 0.8 Hz, 1H), 6.89 (ddd, *J* = 8.3, 2.6, 0.8 Hz, 1H), 3.82 (s, 3H) ppm. The compound was in complete agreement with previously reported data [26,27].

A one-necked round bottom flask was charged with a solution of 1-(2,2-dibromovinyl)-3-methoxybenzene (2.88 g, 9.9 mmol, 1 eq.) in anhydrous tetrahydrofuran (30 mL) and the resulting pale yellow solution was allowed to cool to −78 °C (dry ice/acetone bath) and the mixture was then treated with *n*-butyllithium (15 mL, 21.9 mmol, 2.2 eq., c = 1.47 M) dropwise over 13 min during which time mixture turned brighter yellow, red and finally purple. The mixture was allowed to further stir at −78 °C over 1.5 h. After this time the cooling bath was removed and mixture allowed to stir at ambient temperature for 1.8 h. After this time brown homogeneous mixture was quenched with saturated aq. NH_4_Cl (20 mL) and the mixture was further diluted with H_2_O (20 mL) and Et_2_O (50 mL) and the two layers were well shaken and separated. The aqueous phase was extracted with Et_2_O (2 × 50 mL). The combined organic extracts were washed with brine (40 mL), dried (Na_2_SO_4_) and concentrated in vacuo to give dark yellow oily residue. The residue was purified by chromatography on a silica gel column (eluting with 100% hexanes) to afford the title compound (884 mg, 6.7 mmol, 67%): ^1^H-NMR (400 MHz, CDCl_3_) δ 7.23 (ddm, *J* = 7.5 Hz, 1H), 7.09 (ddd, *J* = 7.6, 1.2 Hz, 1H), 7.02 (dd, *J* = 2.6, 1.4 Hz, 1H), 6.91 (ddd, *J* = 8.3, 2.6, 1.0 Hz, 1H), 3.80 (s, 3H), 3.06 (s, 1H) ppm. The compound was in complete agreement with previously reported data [26,28].

*2-((3-Methoxyphenyl)ethynyl)-6-methylpyridine* (**2**). A two-necked round bottom flask was evacuated, backfilled with an inert atmosphere and then charged with anhydrous *N*,*N*′-dimethylformamide (7 mL) and 2-bromo-6-methylpyridine (0.66 mL, 998 mg, 5.8 mmol, 1 eq., d = 1.512) was added and colourless solution was treated with tetrakis(triphenylphosphine)palladium(0) (201 mg, 0.174 mmol, 0.3 eq.) in one portion and the resulting yellow heterogeneous mixture was allowed to stir at ambient temperature over 13 min. After this time triethylamine (2.42 mL, 1.76 g, 17.4 mmol, 3 eq., d = 0.726) was added and mixture further allowed to stir for 14 min. During this time mixture became completely homogeneous and pale yellow and it was further treated with copper(I)iodide (110 mg, 0.58 mmol, 0.1 eq.) and then a solution of *m*-ethynylanisole (766 mg, 5.80 mmol, 1 eq.) in anhydrous *N*,*N*′-dimethylformamide (7 mL) was added and the resulting green-brown mixture was allowed to stir at ambient temperature over 47.5 h. After this time the mixture was quenched with saturated aq NH_4_Cl (100 mL) and then diluted with EtOAc (150 mL) and the two layers were well shaken and separated. The aqueous phase was extracted with EtOAc (2 × 150 mL). The combined organic extracts were washed with H_2_O (3 × 110 mL), brine (120 mL), dried (Na_2_SO_4_) and concentrated in vacuo. The crude reaction mixture was purified by chromatography on a silica gel column (eluting with a gradient 10% to 20% EtOAc/pentane) to afford the title compound (1.2 g, 5.4 mmol, 93%): ^1^H-NMR (400 MHz, CDCl_3_) δ 7.57 (dd, *J* = 7.7 Hz, 1H), 7.36 (dm, *J* = 7.6 Hz, 1H), 7.26 (dd, *J* = 7.3 Hz, 1H), 7.20 (ddd, *J* = 7.6, 1.3 Hz, 1H, some roofing observed), 7.14 (dd, *J* = 2.6, 1.4 Hz, 1H), 7.11 (dm, *J* = 7.8 Hz, 1H), 6.92 (ddd, *J* = 8.1, 2.6, 1.1 Hz, 1H), 3.82 (s, 3H), 2.59 (s, 3H) ppm. The compound was in complete agreement with previously published data [14].

*2-((3-Methoxyphenyl)ethynyl)-6-methylpyridine hydrochloride salt* (**2**·**HCl**). A one-necked round bottom flask was charged with 2-((3-methoxyphenyl)ethynyl)-6-methylpyridine (108 mg, 0.48 mmol, 1 eq.) and ethanol (1 mL) was added and pale yellow solution was allowed to cool to 0 °C (the ice bath) and it was then treated with ethanolic solution of HCl dopwise over 1 min and the resulting bright yellow solution was allowed to stir at 0 °C for 1 h. After this time the cooling bath was removed and bright yellow mixture was concentrated in vacuo to give crude mixture which was further recrystallized from *^i^*PrOH:EtOH 2:1 to afford the title compound (110 mg, 0.42 mmol, 87%): ^1^H-NMR (400 MHz, CDCl_3_) δ 8.12 (dd, *J* = 7.9 Hz, 1H), 7.66 (dm, *J* = 7.85 Hz, 1H), 7.49–7.45 (m, 2H), 7.43 (ddd, *J* = 7.5, 1.1 Hz, 1H, some roofing observed), 7.31 (ddm, *J* = 8.2 Hz, 1H), 7.02 (ddd, *J* = 8.4, 2.6, 1.0 Hz, 1H), 3.87 (s, 3H), 3.05 (s, 3H) ppm. The compound was in complete agreement with previously published data [14]. 

*4-Bromo-2-methylthiazole* (**8**). A flame dried flask was charged with 2,4-dibromothiazole (500 mg, 2.1 mmol, 1 eq.) and anhydrous diethyl ether was added (12 mL) and the colourless solution was allowed to cool to −78 °C (dry ice/acetone bath) and it was then treated with *n*-butyl lithium (1.6 mL, 2.3 mmol, 1.1 eq., c = 1.47 M) dropwise over 1 min. The mixture turned pale yellow and it was allowed to stir at −78 °C over 79 min. After this time the mixture a solution of dimethyl sulfate (0.6 mL, 779 mg, 6.2 mmol, 3 eq., d = 1.33) in anhydrous diethyl ether (0.5 mL) was added dropwise over 4 min and the resulting mixture allowed to stir at −78 °C over 4 h and then warm to ambient temperature and stir under N_2_ over 15 h. After this time the crude mixture (red in colour) was quenched with saturated NaHCO_3_ (5 mL) and then diluted with H_2_O (8 mL) and EtOAc (20 mL). The two layers were well shaken and separated and the aqueous phase was extracted with EtOAc (2 × 20 mL). The combined organic extracts were washed brine (20 mL), dried (Na_2_SO_4_) and concentrated in vacuo to give crude mixture. The crude mixture was purified by chromatography on a silica gel column (eluting with 10% EtOAc/pentane) to give the title compound (194.3 mg, 1.09 mmol, 53%): ^1^H NMR (400 MHz, CDCl_3_) δ 7.06 (s, 1H), 2.73 (s, 3H) ppm. The compound was in complete agreement with previously published data [29].

*3-((Trimethylsilyl)ethynyl)pyridine* (**17**). A one-necked round bottom flask was charged with 3-ethynyl-pyridine (150 mg, 1.46 mmol, 1 eq.), anhydrous tetrahydrofuran (4.8 mL) was added and pale brown solution was allowed to cool to −78 °C (dry ice/acetone bath) and it was then treated with a solution of lithium hexamethyldisilazide (2 mL, 1.9 mmol, 1.3 eq., c = 1 M) dropwise over 2 min during which time the mixture turned orange and it was allowed to stir at −78 °C for 1 h. After this time orange mixture was treated with trimethylchlorosilane (0.27 mL, 238 mg, 2.19 mmol, 1.5 eq., d = 0.856) and the mixture was allowed to slowly warm to ambient temperature and further stir over 17.5 h. After this time the crude mixture was quenched with H_2_O (10 mL) and then diluted with Et_2_O (10 mL) and the two layers were well shaken and separated. The aqueous phase was further extracted with Et_2_O (2 × 10 mL). The combined organic extracts were washed with brine (10 mL), dried (Na_2_SO_4_) and concentrated in vacuo. The crude mixture was purified by chromatography on a silica gel column (eluting with 5% EtOAc/pentane) to afford the title compound (56.1 mg, 0.32 mmol, 22%): ^1^H-NMR (400 MHz, CDCl_3_) δ 8.69 (dd, *J* = 2.0, 0.7 Hz, 1H), 8.52 (dd, *J* = 4.9, 1.7 Hz, 1H), 7.74 (ddd, *J* = 7.8, 1.9 Hz, 1H), 7.23 (ddd, *J* = 7.9, 4.9, 0.9 Hz, 1H), 0.26 (s, 9H) ppm. The compound was also available from commercial sources and the spectral data were in complete agreement.

*1-Chloro-4-(trimethylsilyl)but-3-yn-2-one* (**19**). A one-necked round bottom flask was charged with aluminium trichloride (5.5 g, 42 mmol, 1.3 eq.) and anhydrous dichloromethane (63 mL) was added and the resulting yellow suspension was allowed to cool to 0 °C (the ice bath). The mixture was then treated with a solution of chloroacetyl chloride (2.6 mL, 3.65 g, 32.3 mmol, 1 eq., d = 1.417) and bis(trimethylsilyl)acetylene (6.6 mL, 5 g, 29.34 mmol, 0.9 eq., d = 0.752) in anhydrous dichloromethane (38 mL) dropwise over 50 min during which time mixture turned darker yellow and finally brown and it was allowed to stir at 0 °C over 1 h. The cooling bath was then removed and the stirring continued at ambient temperature over 65 min. After this time the mixture was allowed to cool to 0 °C (the ice bath) and it was carefully quenched with 1 M aq. HCl (65 mL). The two layers were well shaken and separated. The aqueous phase was further extracted with CH_2_Cl_2_ (2 × 125 mL). The combined organic extracts were washed with H_2_O (125 mL), saturated aq. NaHCO_3_ (125 mL), brine (125 mL), dried (Na_2_SO_4_) and concentrated in vacuo to give brown reside. The crude mixture was purified via Kugelrorh distillation (temperature: 75 °C) at 2 × 10^−2^ kPa to afford the title compound (4.33 g, 24.8 mmol, 77%): ^1^H-NMR (400 MHz, CDCl_3_) δ 4.23 (s, 2H), 0.26 (s, 9H) ppm. The compound was in complete agreement with previously published data [15].

*2-Methyl-4-((trimethylsilyl)ethynyl)thiazole* (**20**). A one-necked round bottom flask was charged with 1-chloro-4-(trimethylsilyl)-3-butyn-2-one (4.3 g, 24.6 mmol, 1 eq.) and anhydrous *N*,*N*′-dimethylformamide (43 mL) was added and the clear yellow solution was treated with thioacetamide (2.4 g, 31.8 mmol, 1.3 eq.) in one portion and the resulting yellow homogeneous mixture was allowed to stir at ambient temperature over 17 h. After this time the crude mixture was diluted with EtOAc (200 mL) and the organic phase was washed with H_2_O (3 × 150 mL), brine (150 mL), dried (Na_2_SO_4_) and concentrated in vacuo to give brown oily residue. The crude mixture was purified by chromatography on a silica gel column (eluting with gradient 2% to 4% EtOAc/hexanes) to afford the title compound (4.75 g, 24.3 mmol, 99%): ^1^H-NMR (400 MHz, CDCl_3_) δ 7.32 (s, 1H), 2.70 (s, 3H), 0.24 (s, 9H) ppm. The compound was in complete agreement with previously published data [15].

*4-Ethynyl-2-methylthiazole* (**10**). A one-necked round bottom flask was charged with 2-methyl-4-((trimethylsilyl)ethynyl)thiazole (400 mg, 2.05 mmol, 1 eq.) and methanol (0.5 mL) was added and the red mixture was further treated with a solution of potassium hydroxide (230 mg, 4.1 mmol, 2 eq.) in methanol (4.8 mL) in one portion and the resulting dark brown mixture was allowed to stir over 3.5 h. After this time the mixture was quenched with H_2_O (10 mL) and diluted with EtOAc (10 mL) and the two layers were well shaken and separated. The aqueous phase was extracted with EtOAc (3 × 8 mL). The combined organic extracts were washed with brine (8 mL), dried (Na_2_SO_4_) and concentrated in vacuo to give crude mixture. The crude mixture was purified by chromatography on a silica gel column (eluting with gradient 5% to 10% EtOAc/pentane) to afford the title compound (172.6 mg, 1.40 mmol, 68%): ^1^H-NMR (400 MHz, CDCl_3_) δ 7.37 (s, 1H), 3.09 (s, 1H), 2.71 (s, 3H) ppm. The compound was also available from commercial sources and the spectral data were in complete agreement.

*2-Methyl-4-(pyridin-3-ylethynyl)thiazole* (**3**). A one-necked round bottom flask was charged with 2-methyl-4-[(trimethylsilyl)ethynyl]-1,3-thiazole (3.84 g, 19.7 mmol, 1 eq.) and 3-bromopyridine (2.1 mL, 3.42 g, 21.6 mmol, 1.1 eq., d = 1.64) was added in one portion and then 1,2-dimethoxyethane (50 mL) was added and the resulting brown heterogeneous mixture was treated with triethylamine (5.5 mL, 3.98 g, 39.4 mmol, 2 eq.) in one portion and the mixture was sparged with N_2_ and the flask was allowed to heat (temperature of preheated oil bath: 70 °C). Immediately upon heating tetrakis(triphenylphosphine)palladium (0) (446 mg, 0.39 mmol, 0.02 eq.) was added and sparging continued for another 14 min. After this time sparging was discontinued and the mixture treated with a solution of tetrabutylammonium fluoride (25 mL, 25.4 mmol, 1.3 eq., c = 1 M) in tetrahydrofuran was added via syringe pump (5 mL/h in 20 mL syringe) whilst the mixture was heated over 20 h. The crude mixture was concentrated in vacuo and the residue dissolved in EtOAc (400 mL) and the organic phase washed with H_2_O (200 mL). The organic layer was dried (Na_2_SO_4_) and concentrated in vacuo to give brown oily residue. The crude reaction mixture was purified by chromatography on a silica gel column (eluting with gradient 30% to 50% EtOAc/hexanes) to give the title compound (2.06 g, 10.3 mmol, 52%). The material was then recrystallized from hot EtOAc layered with cold hexanes to afford yellow needles (1.32 g, 6.6 mmol, 33%): ^1^H-NMR (400 MHz, CDCl_3_) δ 8.79 (bd, *J* = 1.3 Hz, 1H), 8.57 (dd, *J* = 4.8, 1.4 Hz, 1H), 7.83 (ddd, *J* = 7.9, 1.9 Hz, 1H), 7.43 (s, 1H), 7.29 (ddd, *J* = 7.8, 4.9, 0.8 Hz, 1H), 2.75 (s, 3H) ppm. The compound was in complete agreement with previously published data [15,17].

*2-Methyl-4-(pyridin-3-ylethynyl)thiazole hydrochloride salt* (**3·HCl**). A one-necked round bottom flask was charged with 2-methyl-4-(pyridin-3-ylethynyl)thiazole (214 mg, 1.07 mmol, 1 eq.) and ethanolic solution of hydrochloric acid (1.1 mL, 1.07 mmol, 1 eq., c = 1 M) was added but material did not completely dissolve and additional EtOH (1 mL) was added and the resulting heterogeneous mixture allowed to stir at ambient temperature over 30 min. After this time the mixture was concentrated in vacuo and the residue recrystallized from *^i^*PrOH to yield the title compound (168.3 mg, 0.71 mmol, 66%): ^1^H-NMR (400 MHz, CDCl_3_) δ 8.87 (bs, 1H), 8.74 (bd, *J* = 5.4 Hz, 1H), 8.46 (bddd, *J* = 8.1, 1.6 Hz, 1H), 7.92 (bdd, *J* = 8.0, 1.6 Hz, 1H), 7.63 (s, 1H), 2.77 (s, 3H) ppm. The compound was also available from commercial sources and the spectral data were in complete agreement.

*2-Ethynyl-6-methylpyridine* (**6**). A solution of 2-bromo-6-methylpyridine (700 mg, 4.06 mmol, 1 eq.) in triethylamine (degassed, 11.7 mL) was at ambient temperature treated with trimethylsilylacetylene (0.63 mL, 438 mg, 4.47 mmol, 1.1 eq., d = 0.709), copper(I)iodide (76 mg, 0.4 mmol, 0.1 eq.) and *trans*-dichlorobis(triphenylphospine)palladium (280 mg, 0.4 mmol, 0.1 eq.). The resulting solution was allowed to stir at ambient temperature over 17 h. After this time the crude mixture was quenched with H_2_O (8 mL) and further extracted with EtOAc (3 × 10 mL). The combined organic extracts were dried (Na_2_SO_4_) and concentrated in vacuo and the crude mixture was purified by chromatography on a silica gel column (eluting with 5% Et_2_O/pentane) to afford 2-methyl-6-((trimethylsilyl)-ethynyl)pyridine (537 mg, 2.84 mmol, 70%): ^1^H-NMR (400 MHz, CDCl_3_) δ 7.52 (dd, *J* = 7.7 Hz, 1H), 7.28 (dm, *J* = 7.9 Hz, 1H), 7.09 (dm, *J* = 7.6 Hz, 1H), 2.55 (s, 3H), 0.26 (s, 9H) ppm. The compound was in complete agreement with previously published data [14]. 

A yellow solution of 2-methyl-6-((trimethylsilyl)ethynyl)pyridine (226 mg, 1.19 mmol, 1 eq.) in methanol (0.3 mL) was treated with the solution of potassium hydroxide (134 mg, 2.39 mmol, 2 eq.) in methanol (2.5 mL) and the resulting colourless solution was allowed to stir at ambient temperature over 2.5 h. After this time the crude mixture was quenched with H_2_O (6 mL) and diluted with EtOAc (5 mL) and the two layers were well shaken and separated. The aqueous phase was extracted with EtOAc (3 × 5 mL). The combined organic extracts were washed with brine (3 mL), dried (Na_2_SO_4_) and concentrated in vacuo. The crude reaction mixture was purified by chromatography on a silica gel column (eluting with gradient 5% to 10% EtOAc/pentane) to afford the title compound (84 mg, 0.72 mmol, 60%): ^1^H-NMR (400 MHz, CDCl_3_) δ 7.54 (dd, *J* = 7.8 Hz, 1H), 7.29 (dm, *J* = 7.8 Hz, 1H), 7.13 (dm, *J* = 7.8 Hz, 1H), 3.12 (s, 1H), 2.55 (s, 3H) ppm. The compound was in complete agreement with previously published data [14]. 

## 4. Conclusions

In conclusion, employing Sonogashira cross-coupling reaction, two mGlu_5_ antagonists MMPEP (**2**) and MTEP (**3**) were prepared by an improved synthetic method in 62% and 40% overall yields, respectively. Computationally calculated HOMO energies for the reacting partners suggested that product formation is controlled by both reacting partners, the halide and the alkyne. Our findings revealed that synthetically meaningful yields were achieved when HOMO energy of the halide was lower than that of the alkyne. Furthermore, our results point to possibility of other high-lying molecular orbitals which may be more critical predictors of reactivity. While not all halide-alkyne combinations were attempted, the experimental data of our study support the computationally obtained conclusions, thus furthering our understanding of the requirements for the successful Sonogashira cross-coupling.

## Supplementary Materials

The following are available online at http://www.mdpi.com/1424-8247/11/1/24/s1, detailed experimental procedure and characterization data as well as Cartesian co-ordinates for all reported compounds.
