# Supplementary material for: Improved Syntheses of the mGlu5 Antagonists MMPEP and MTEP Using Sonogashira Cross-Coupling
Source: Pharmaceuticals (Basel). 2018 Feb 20;11(1):24. doi: 10.3390/ph11010024 (PMC5874720; doi:10.3390/ph11010024)
Supplement: Supplementary file 1 [file pharmaceuticals-11-00024-s001.pdf]

Supporting information: CONTENTS

Improved Syntheses of mGlu$_5$ Antagonists MMPEP and MTEP Using Sonogashira Cross-Coupling

Boshuai Mu, Linjing Mu, Roger Schibli, Simon M. Ametamey and Selena Milicevic Sephton*

Experimental procedures S2-S20

General techniques S2
compound **S1** S3
compound **5A** S4
compound **2**, MMPEP S5
compound **2·HCl** S6
compound **8** S6/S7
compound **9** S7
compound **3**, MTEP S8/S12/S16/S17
compound **17** S9
compound **19** S10
compound **20** S11
compound **10** S11/S12/S15
compound **3·HCl** S13
compound **S2** S13/S14
compound **6** S14/S15

References S17

^{1}H NMR Spectra S18-S31

Computational evaluation S32-S43

compound **4** S33
compound **21** S34
compound **11** S35
compound **7A** S36
compound **8** S37
compound **5A** S38
compound **6** S39
compound **9** S40
compound **10** S41
compound **15** S42
compound **22** S43

Supporting Information: Experimental Procedures

Improved Syntheses of mGlu$_5$ Antagonists MMPEP and MTEP Using Sonogashira Cross-Coupling

Boshuai Mu, Linjing Mu, Roger Schibli, Simon M. Ametamey and Selena Milicevic Sephton*

Experimental procedures

General techniques: All reactions requiring anhydrous conditions were conducted in flame-dried glass apparatus under an atmosphere of inert gas. All chemicals and anhydrous solvents were purchased from Aldrich or ABCR and used as received unless otherwise noted. Reported density values are for ambient temperature.

Preparative chromatographic separations were performed on Aldrich Science silica gel 60 (35-75 μm) and reactions followed by TLC analysis using Sigma-Aldrich silica gel 60 plates (2-25 μm) with fluorescent indicator (254 nm) and visualized with UV or potassium permanganate.

^{1}H and ^{13}C NMR spectra were recorded in Fourier transform mode at the field strength specified on Bruker Avance FT-NMR spectrometers. Spectra were obtained from the specified deuterated solvents in 5 mm diameter tubes. Chemical shift in ppm is quoted relative to residual solvent signals calibrated as follows: **$CDCl_3$** δ_H ($CHCl_3$) = 7.26 ppm, δ_C = 77.2 ppm. Multiplicities in the ^{1}H NMR spectra are described as: s = singlet, d = doublet, t = triplet, q = quartet, quint. = quintet, m = multiplet, b = broad; coupling constants are reported in Hz.

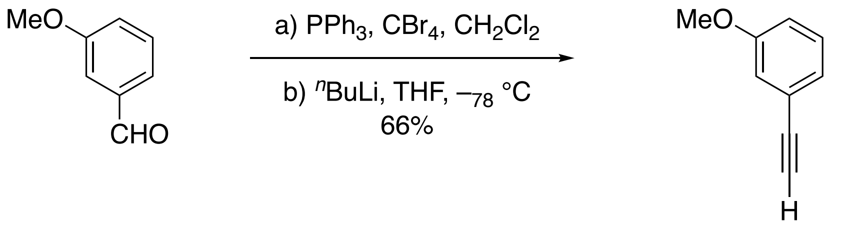

14 $C_8H_8O_2$ (136.15) **5A** C_9H_8O (132.16)

1-(2,2-Dibromovinyl)-3-methoxybenzene (S1)

One neck round bottom flask was charged with triphenylphosphine (10.5 g, 40 mmol, 4 eq), then carbontetrabromide (6.63 g, 20 mmol, 2 eq) and the yellow solid mixture was carefully dissolved in anhydrous dichloromethane (36 mL; CAUTION: vigorous reaction!) and the resulting orange mixture was allowed to cool to 0 °C (the ice bath). The heterogeneous and red in colour mixture was allowed to stir and then treated with *m*-anisaldehyde (1.2 mL, 1.36 g, 10 mmol, 1 eq, d=1.119) dropwise over 1 min and the resulting dark orange mixture was allowed to stir at 0 °C for 30 min and then the cooling bath was removed and stirring continued at ambient temperature for 38 min. After this time the crude mixture was quenched with ice cold H_2O (40 mL) and diluted with hexanes (25 mL) and the two layers were well shaken and separated. The aqueous phase was extracted with hexanes (5x25 mL). The combined organic extracts were concentrated *in vacuo* and the crude mixture was purified by chromatography on a silica gel column (eluting with 100% hexanes) to afford the title compound (2.88 g, 9.9 mmol, 99%): ^{1}H NMR (400 MHz, $CDCl_3$) δ 7.46 (bs, 1H), 7.29 (t, J = 8.0 Hz, 1H), 7.12 (tm, J = 1.9 Hz, 1H), 7.09 (ddt, J = 7.7, 1.4, 0.8 Hz, 1H), 6.89 (ddd, J = 8.3, 2.6, 0.8 Hz, 1H), 3.82 (s, 3H) ppm. The compound was in complete agreement with previously reported data.[1,2]

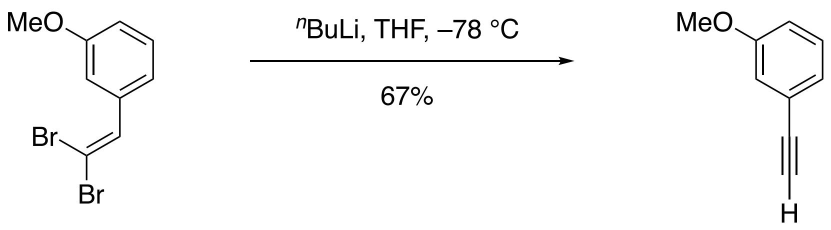

S1
$C_9H_8Br_2O$
(291.97)

5A
C_9H_8O
(132.16)

1-Ethynyl-3-methoxybenzene (5A)

One neck round bottom flask was charged with a solution of 1-(2,2-dibromovinyl)-3-methoxybenzene (2.88 g, 9.9 mmol, 1eq) in anhydrous tetrahydrofuran (30 mL) and the resulting pale yellow solution was allowed to cool to –78 °C (dry ice/acetone bath) and the mixture was then treated with *n*-butyllithium (15 mL, 21.9 mmol, 2.2 eq, c=1.47 M) dropwise over 13 min during which time mixture turned brighter yellow, red and finally purple. The mixture was allowed to further stir at –78 °C over 1.5 h. After this time the cooling bath was removed and mixture allowed to stir at ambient temperature for 1.8 h. After this time brown homogeneous mixture was quenched with saturated aq. NH_4Cl (20 mL) and the mixture was further diluted with H_2O (20 mL) and Et_2O (50 mL) and the two layers were well shaken and separated. The aqueous phase was extracted with Et_2O (2x50 mL). The combined organic extracts were washed with brine (40 mL), dried (Na_2SO_4) and concentrated *in vacuo* to give dark yellow oily residue. The residue was purified by chromatography on a silica gel column (eluting with 100% hexanes) to afford the title compound (884 mg, 6.7 mmol, 67%): ^{1}H NMR (400 MHz, $CDCl_3$) δ 7.23 (ddm, J = 7.5 Hz, 1H), 7.09 (ddd, J = 7.6, 1.2 Hz, 1H), 7.02 (dd, J = 2.6, 1.4 Hz, 1H), 6.91 (ddd, J = 8.3, 2.6, 1.0 Hz, 1H), 3.80 (s, 3H), 3.06 (s, 1H) ppm. The compound was in complete agreement with previously reported data.[1,3]

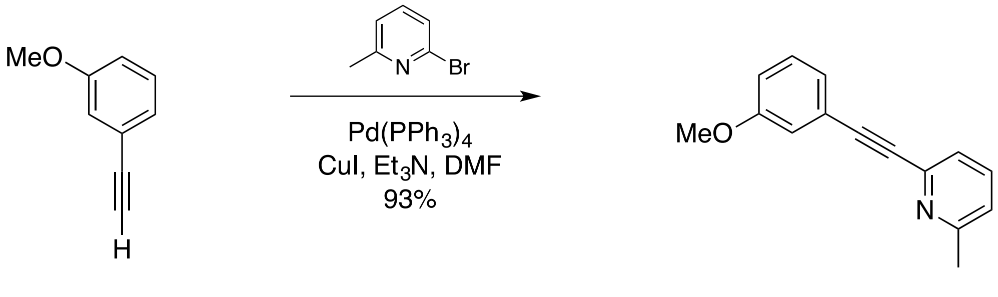

5A
C_9H_8O
(132.16)

2, MMPEP
$C_{15}H_{13}NO$
(223.28)

2-((3-Methoxyphenyl)ethynyl)-6-methylpyridine (2)

Two neck round bottom flask was evacuated and backfilled with inert atmosphere and then charged with anhydrous *N,N′*-dimethylformamide (7 mL) and 2-bromo-6-methylpyridine (0.66 mL, 998 mg, 5.8 mmol, 1 eq, d=1.512) was added and colourless solution was treated with tetrakis(triphenylphosphine)palladium(0) (201 mg, 0.174 mmol, 0.3 eq) in one portion and the resulting yellow heterogeneous mixture was allowed to stir at ambient temperature over 13 min. After this time triethylamine (2.42 mL, 1.76 g, 17.4 mmol, 3 eq, d=0.726) was added and mixture further allowed to stir for 14 min. During this time mixture became completely homogeneous and pale yellow and it was further treated with copper(I)iodide (110 mg, 0.58 mmol, 0.1 eq) and then a solution of *m*-ethynylanisole (766 mg, 5.80 mmol, 1 eq) in anhydrous *N,N′*-dimethylformamide (7 mL) was added and the resulting green-brown mixture was allowed to stir at ambient temperature over 47.5 h. After this time the mixture was quenched with saturated aq NH_4Cl (100 mL) and then diluted with EtOAc (150 mL) and the two layers were well shaken and separated. The aqueous phase was extracted with EtOAc (2x150 mL). The combined organic extracts were washed with H_2O (3x110 mL), brine (120 mL), dried (Na_2SO_4) and concentrated *in vacuo*. The crude reaction mixture was purified by chromatography on a silica gel column (eluting with a gradient 10% to 20% EtOAc/pentane) to afford the title compound (1.2 g, 5.4 mmol, 93%): ^{1}H NMR (400 MHz, $CDCl_3$) δ 7.57 (dd, *J* = 7.7 Hz, 1H), 7.36 (dm, *J* = 7.6 Hz, 1H), 7.26 (dd, *J* = 7.3 Hz, 1H), 7.20 (ddd, *J* = 7.6, 1.3 Hz, 1H, some roofing observed), 7.14 (dd, *J* = 2.6, 1.4 Hz, 1H), 7.11 (dm, *J* = 7.8 Hz, 1H), 6.92 (ddd, *J* = 8.1, 2.6, 1.1 Hz, 1H), 3.82 (s, 3H), 2.59 (s, 3H) ppm. The compound was in complete agreement with previously published data.[4]

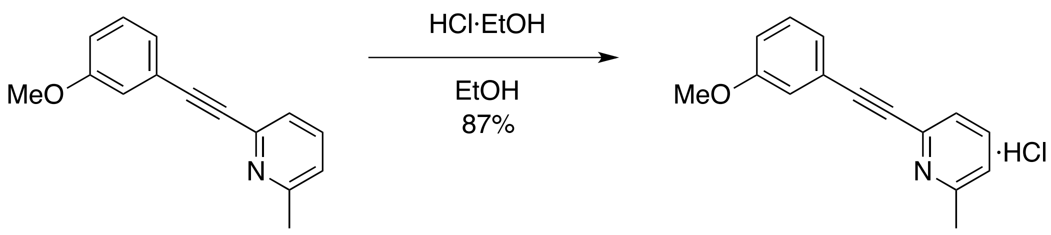

2, MMPEP
$C_{15}H_{13}NO$
(223.28)

2·HCl, MMPEP·HCl
$C_{15}H_{14}ClNO$
(259.73)

2-((3-Methoxyphenyl)ethynyl)-6-methylpyridine hydrochloride salt (2·HCl)

One neck round bottom flask was charged with 2-((3-methoxyphenyl)ethynyl)-6-methylpyridine (108 mg, 0.48 mmol, 1 eq) and ethanol (1 mL) was added and pale yellow solution was allowed to cool to 0 °C (the ice bath) and it was then treated with ethanolic solution of HCl dopwise over 1 min and the resulting bright yellow solution was allowed to stir at 0 °C for 1 h. After this time the cooling bath was removed and bright yellow mixture was concentrated *in vacuo* to give crude mixture which was further recrystallized from ^{i}PrOH:EtOH 2:1 to afford the title compound (110 mg, 0.42 mmol, 87%): ^{1}H NMR (400 MHz, $CDCl_3$) δ 8.12 (dd, *J* = 7.9 Hz, 1H), 7.66 (dm, *J* = 7.85 Hz, 1H), 7.49-7.45 (m, 2H), 7.43 (ddd, *J* = 7.5, 1.1 Hz, 1H, some roofing observed), 7.31 (ddm, *J* = 8.2 Hz, 1H), 7.02 (ddd, *J* = 8.4, 2.6, 1.0 Hz, 1H), 3.87 (s, 3H), 3.05 (s, 3H) ppm. The compound was in complete agreement with previously published data.[4]

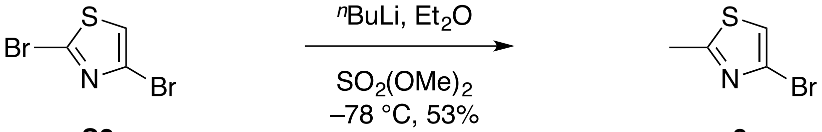

S2
C_3HBr_2NS
(242.92)

8
C_4H_4BrNS
(178.05)

4-Bromo-2-methylthiazole (8)

A flame dried flask was charged with 2,4-dibromothiazole (500 mg, 2.1 mmol, 1 eq) and anhydrous diethylether was added (12 mL) and the colourless solution was allowed to cool to –78 °C (dry ice/acetone bath) and it was then treated with *n*-butyl lithium (1.6 mL, 2.3 mmol, 1.1 eq, c=1.47 M) dropwise over 1 min. The mixture turned pale yellow and it was allowed to stir at –78 °C over 79 min. After this time the mixture a solution of dimethylsulfate (0.6 mL, 779 mg, 6.2 mmol, 3 eq, d=1.33) in anhydrous diethylether (0.5 mL) was added dropwise over 4 min and the resulting mixture allowed to stir at –78 °C over 4 h and then warm to

ambient temperature and stir under N_2 over 15 h. After this time the crude mixture (red in colour) was quenched with saturated $NaHCO_3$ (5 mL) and then diluted with H_2O (8 mL) and EtOAc (20 mL). The two layers were well shaken and separated and the aqueous phase was extracted with EtOAc (2x20 mL). The combined organic extracts were washed brine (20 mL), dried (Na_2SO_4) and concentrated *in vacuo* to give crude mixture. The crude mixture was purified by chromatography on a silica gel column (eluting with 10% EtOAc/pentane) to give the title compound (194.3 mg, 1.09 mmol, 53%): ^1H NMR (400 MHz, $CDCl_3$) δ 7.06 (s, 1H), 2.73 (s, 3H) ppm. The compound was in complete agreement with previously published data.[5]

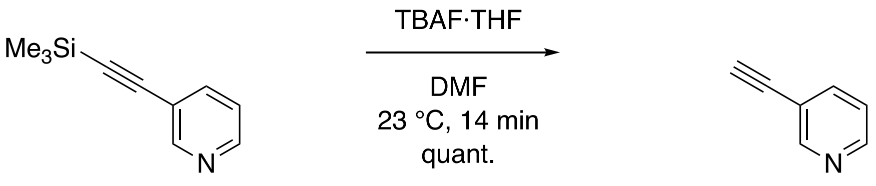

17
$C_{10}H_{13}NSi$
(175.731)

9
C_7H_5N
(103.12)

3-Ethynylpyridine (9)

One neck round bottom flask was charged with 3-((trimethylsilyl)ethynyl)pyridine (44 mg, 0.25 mmol, 1 eq), and anhydrous *N,N′*-dimethylformamide (1 mL) was added and the clear homogeneous solution was further treated with tetrabutylammonium fluoride solution in tetrahydrofuran (0.5 mL, 0.5 mmol, 2eq, c=1 M) dropwise (<1 min) and the resulting brown mixture was allowed to stir at ambient temperature under nitrogen atmosphere over 14 min. This material without work-up or purification was used for the next step.

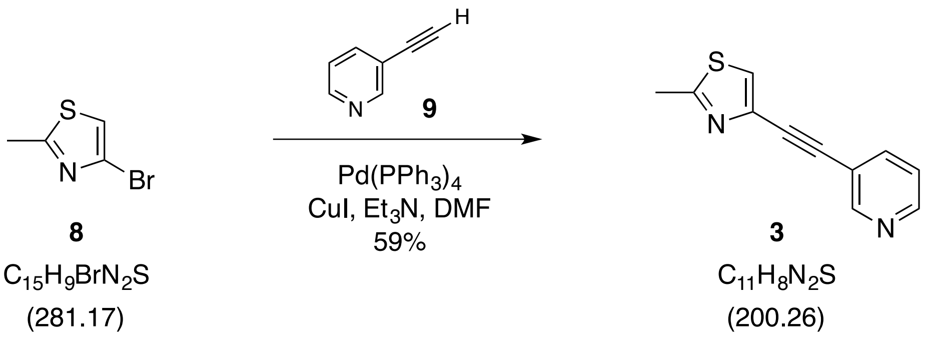

2-Methyl-4-(pyridin-3-ylethynyl)thiazole (3)

A two neck round bottom flask was evacuated and then backfilled with nitrogen atmosphere and this was repeated two more times. This flask was then charged with a solution of 4-bromo-2-methylthiazole (194 mg, 1.09 mmol, 1 eq) in anhydrous *N*,*N′*-dimethylformamide (1.5 mL) prepared in separate flame dried flask under inert atmosphere. To this solution was then added tetrakis(triphenylphosphine)palladium (0) (38 mg, 0.033 mmol, 0.03 eq) in one portion and brown mixture was allowed to stir for 9 min. After this time triethylamine (0.45 mL, 330 mg, 3.27 mmol, 3 eq, d=0.726) was added and mixture allowed to stir further over 12 min. After this time, still heterogeneous mixture, was treated with copper(I)iodide (21 mg, 0.11 mmol, 0.1 eq) after which it turned dark brown. Finally, a solution of 3-ethynylpyridine (112 mg, 1.09 mmol, 1 eq) in anhydrous *N*,*N′*-dimethylformamide (1.5 mL) was added and the mixture allowed to stir at ambient temperature over 25 h. After this time the reaction mixture was quenched with saturated aq, NH_4Cl (20 mL) and diluted with EtOAc (30 mL) and the two layers were well shaken and separated. The aqueous phase was extracted with EtOAc (3x30 mL). The combined organic extracts were washed with H_2O (3x25 mL), brine (25 mL), dried (Na_2SO_4) and concentrated *in vacuo*. The crude mixture was purified by chromatography on a silica gel column (eluting with gradient 10% EtOAc/pentane to 100% EtOAc) to afford the inseparable mixture (59 mg,). The NMR analysis revealed 8% conversion to the title compound.

Note: When the reaction was repeated it failed to yield desired product.

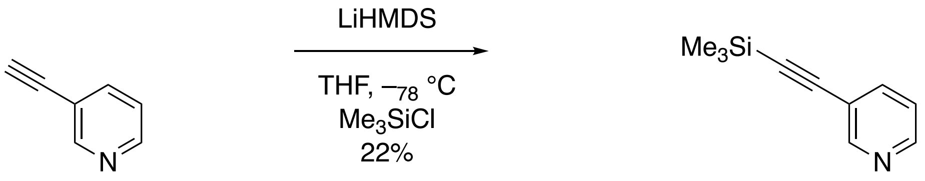

9
C_7H_5N
(103.12)

17
$C_{10}H_{13}NSi$
(175.731)

3-((Trimethylsilyl)ethynyl)pyridine (17)

One neck round bottom flask was charged with 3-ethynylpyridine (150 mg, 1.46 mmol, 1 eq) and anhydrous tetrahydrofuran (4.8 mL) was added and pale brown solution was allowed to cool to –78 °C (dry ice/acetone bath) and it was then treated with a solution of lithiumhexamethyldisilazide (2 mL, 1.9 mmol, 1.3 eq, c = 1M) dropwise over 2 min during which time the mixture turned orange and it was allowed to stir at –78 °C for 1 h. After this time orange mixture was treated with trimethylchlorosilane (0.27 mL, 238 mg, 2.19 mmol, 1.5 eq, d=0.856) and the mixture was allowed to slowly warm to ambient temperature and further stir over 17.5 h. After this time the crude mixture was quenched with H_2O (10 mL) and then diluted with Et_2O (10 mL) and the two layers were well shaken and separated. The aqueous phase was further extracted with Et_2O (2x10 mL). The combined organic extracts were washed with brine (10 mL), dried (Na_2SO_4) and concentrated *in vacuo*. The crude mixture was purified by chromatography on a silica gel column (eluting with 5% EtOAc/pentane) to afford the title compound (56.1 mg, 0.32 mmol, 22%): ^{1}H NMR (400 MHz, $CDCl_3$) δ 8.69 (dd, *J* = 2.0, 0.7 Hz, 1H), 8.52 (dd, *J* = 4.9, 1.7 Hz, 1H), 7.74 (ddd, *J* = 7.8, 1.9 Hz, 1H), 7.23 (ddd, *J* = 7.9, 4.9, 0.9 Hz, 1H), 0.26 (s, 9H) ppm. The compound was also available from commercial sources and the spectral data were in complete agreement.

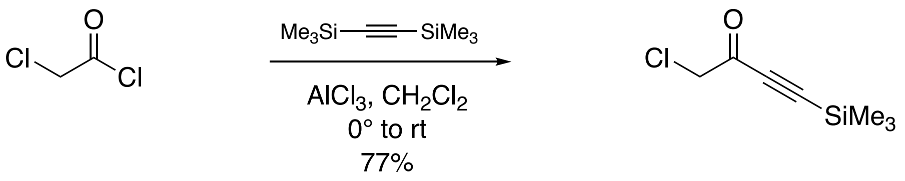

18
$C_2H_2Cl_2O$
(112.94)

19
$C_7H_{11}ClOSi$
(174.70)

1-Chloro-4-(trimethylsilyl)but-3-yn-2-one (19)

One neck round bottom flask was charged with aluminium trichloride (5.5 g, 42 mmol, 1.3 eq) and anhydrous dichloromethane (63 mL) was added and the resulting yellow suspension was allowed to cool to 0 °C (the ice bath). The mixture was then treated with a solution of chloroacetylchloride (2.6 mL, 3.65 g, 32.3 mmol, 1 eq, d=1.417) and bis(trimethylsilyl)acetylene (6.6 mL, 5 g, 29.34 mmol, 0.9 eq, d=0.752) in anhydrous dichloromethane (38 mL) dropwise over 50 min during which time mixture turned darker yellow and finally brown and it was allowed to stir at 0 °C over 1 h. The cooling bath was then removed and the stirring continued at ambient temperature over 65 min. After this time the mixture was allowed to cool to 0 °C (the ice bath) and it was carefully quenched with 1M aq. HCl (65 mL). The two layers were well shaken and separated. The aqueous phase was further extracted with CH_2Cl_2 (2x125 mL). The combined organic extracts were washed with H_2O (125 mL), saturated aq. $NaHCO_3$ (125 mL), brine (125 mL), dried (Na_2SO_4) and concentrated *in vacuo* to give brown reside. The crude mixture was purified *via* Kugelrorh distillation (temperature: 75 °C) at $2x10^{-2}$ kPa to afford the title compound (4.33 g, 24.8 mmol, 77%): ^1H NMR (400 MHz, $CDCl_3$) δ 4.23 (s, 2H), 0.26 (s, 9H) ppm. The compound was in complete agreement with previously published data.[6]

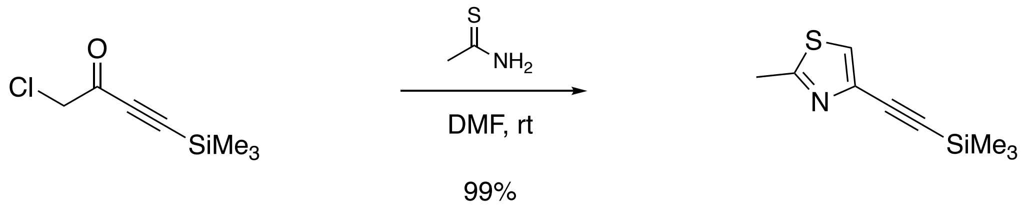

19
$C_7H_{11}ClOSi$
(174.70)

20
$C_9H_{13}NSSi$
(195.36)

2-Methyl-4-((trimethylsilyl)ethynyl)thiazole (20)

One neck round bottom flaks was charged with 1-chloro-4-(trimethylsilyl)-3-butyn-2-one (4.3 g, 24.6 mmol, 1 eq) and anhydrous *N,N′*-dimethylformamide (43 mL) was added and the clear yellow solution was treated with thioacetamide (2.4 g, 31.8 mmol, 1.3 eq) in one portion and the resulting yellow homogeneous mixture was allowed to stir at ambient temperature over 17 h. After this time the crude mixture was diluted with EtOAc (200 mL) and the organic phase was washed with H_2O (3x150 mL), brine (150 mL), dried (Na_2SO_4) and concentrated *in vacuo* to give brown oily residue. The crude mixture was purified by chromatography on a silica gel column (eluting with gradient 2% to 4% EtOAc/hexanes) to afford the title compound (4.75 g, 24.3 mmol, 99%): ^{1}H NMR (400 MHz, $CDCl_3$) δ 7.32 (s, 1H), 2.70 (s, 3H), 0.24 (s, 9H) ppm. The compound was in complete agreement with previously published data.[6]

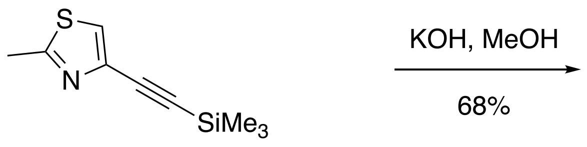

20
$C_9H_{13}NSSi$
(195.36)

10
C_6H_5NS
(123.17)

4-Ethynyl-2-methylthiazole (10)

One neck round bottom flask was charged with 2-methyl-4-((trimethylsilyl)ethynyl)thiazole (400 mg, 2.05 mmol, 1 eq) and methanol (0.5 mL) was added and the red mixture was further treated with a solution of potassium hydroxide (230 mg, 4.1 mmol, 2 eq) in methanol (4.8 mL) in one portion and the resulting dark brown mixture was allowed to stir over 3.5 h. After this time the mixture was quenched with H_2O (10 mL) and diluted with EtOAc (10 mL) and the two layers were well shaken and separated. The aqueous phase was extracted with EtOAc

(3x8 mL). The combined organic extracts were washed with brine (8 mL), dried (Na_2SO_4) and concentrated *in vacuo* to give crude mixture. The crude mixture was purified by chromatography on a silica gel column (eluting with gradient 5% to 10% EtOAc/pentane) to afford the title compound (172.6 mg, 1.40 mmol, 68%): ^{1}H NMR (400 MHz, $CDCl_3$) δ 7.37 (s, 1H), 3.09 (s, 1H), 2.71 (s, 3H) ppm. The compound was also available from commercial sources and the spectral data were in complete agreement.

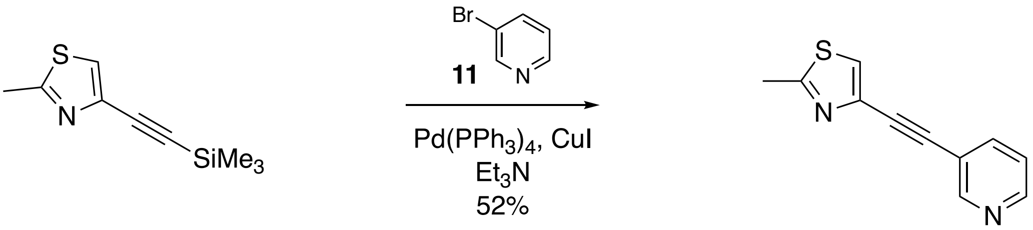

20
$C_9H_{13}NSSi$
(195.36)

3, MTEP
$C_{11}H_8N_2S$
(200.26)

2-Methyl-4-(pyridin-3-ylethynyl)thiazole (3)

One neck round bottom flask was charged with 2-methyl-4-[(trimethylsilyl)ethynyl]-1,3-thiazole (3.84 g, 19.7 mmol, 1 eq) and 3-bromopyridine (2.1 mL, 3.42 g, 21.6 mmol, 1.1 eq, d=1.64) was added in one portion and then 1,2-dimethoxyethane (50 mL) was added and the resulting brown heterogeneous mixture was treated with triethylamine (5.5 mL, 3.98 g, 39.4 mmol, 2 eq) in one portion and the mixture was sparged with N_2 and the flask was allowed to heat (temperature of preheated oil bath: 70 °C). Immediately upon heating tetrakis(triphenylphosphine)palladium (0) (446 mg, 0.39 mmol, 0.02 eq) was added and sparging continued for another 14 min. After this time sparging was discontinued and the mixture treated with a solution of tetrabutylammonium fluoride (25 mL, 25.4 mmol, 1.3 eq, c = 1M) in tetrahydrofuran was added *via* syringe pump (5 mL/hour in 20 mL syringe) whilst the mixture was heated over 20 h. The crude mixture was concentrated *in vacuo* and the residue dissolved in EtOAc (400 mL) and the organic phase washed with H_2O (200 mL). The organic layer was dried (Na_2SO_4) and concentrated *in vacuo* to give brown oily residue. The crude reaction mixture was purified by chromatography on a silica gel column (eluting with gradient 30% to 50% EtOAc/hexanes) to give the title compound (2.06 g, 10.3 mmol, 52%). The material was then recrystallized from hot EtOAc layered with cold hexanes to afford yellow needles (1.32 g, 6.6 mmol, 33%): ^{1}H NMR (400 MHz, $CDCl_3$) δ 8.79 (bd, J = 1.3 Hz, 1H), 8.57 (dd, J = 4.8, 1.4 Hz, 1H), 7.83 (ddd, J = 7.9, 1.9 Hz, 1H), 7.43 (s, 1H), 7.29 (ddd, J = 7.8, 4.9, 0.8 Hz, 1H), 2.75 (s, 3H) ppm. The compound was in complete agreement with previously published data.[6,7]

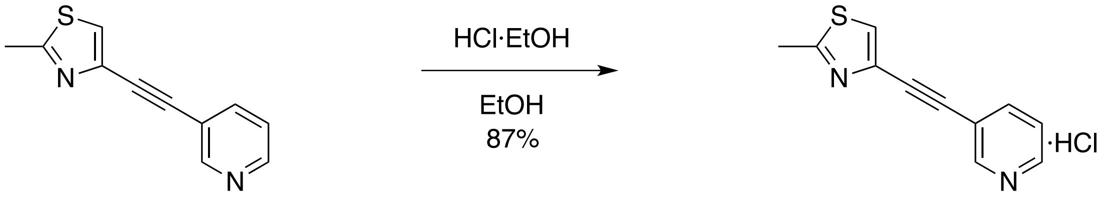

3, MTEP
$C_{11}H_8N_2S$
(200.26)

3·HCl, MTEP·HCl
$C_{11}H_9ClN_2S$
(236.72)

2-Methyl-4-(pyridin-3-ylethynyl)thiazole hydrochloride salt (3·HCl)

One neck round bottom flask was charged with 2-methyl-4-(pyridin-3-ylethynyl)thiazole (214 mg, 1.07 mmol, 1 eq) and ethanolic solution of hydrochloric acid (1.1 mL, 1.07 mmol, 1 eq, c = 1M) was added but material did not completely dissolve and additional EtOH (1 mL) was added and the resulting heterogeneous mixture allowed to stir at ambient temperature over 30 min. After this time the mixture was concentrated *in vacuo* and the residue recrystallized from ^{i}PrOH to yield the title compound (168.3 mg, 0.71 mmol, 66%): ^{1}H NMR (400 MHz, $CDCl_3$) δ 8.87 (bs, 1H), 8.74 (bd, J = 5.4 Hz, 1H), 8.46 (bddd, J = 8.1, 1.6 Hz, 1H), 7.92 (bdd, J = 8.0, 1.6 Hz, 1H), 7.63 (s, 1H), 2.77 (s, 3H) ppm. The compound was also available from commercial sources and the spectral data were in complete agreement.

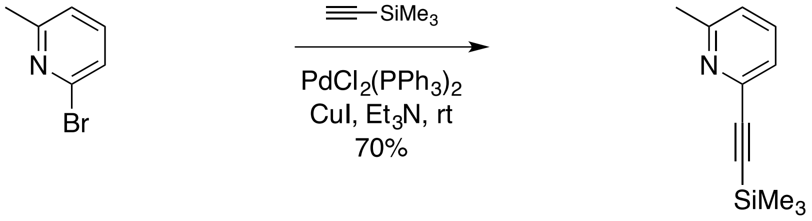

4
C_6H_6BrN
(172.02)

S2
$C_{11}H_{15}NSi$
(189.33)

2-Methyl-6-((trimethylsilyl)ethynyl)pyridine (S2)

A solution of 2-bromo-6-methylpyridine (700 mg, 4.06 mmol, 1 eq) in triethylamine (degassed, 11.7 mL) was at ambient temperature treated with trimethylsilylacetylene (0.63 mL, 438 mg, 4.47 mmol, 1.1 eq, d=0.709), copper(I)iodide (76 mg, 0.4 mmol, 0.1 eq) and *trans*-dichlorobis(triphenylphospine)palladium (280 mg, 0.4 mmol, 0.1 eq). The resulting

solution was allowed to stir at ambient temperature over 17 h. After this time the crude mixture was quenched with H_2O (8 mL) and further extracted with EtOAc (3x10 mL). The combined organic extracts were dried (Na_2SO_4) and concentrated *in vacuo* and the crude mixture was purified by chromatography on a silica gel column (eluting with 5% Et_2O/pentane) to afford the title compound (537 mg, 2.84 mmol, 70%): ^{1}H NMR (400 MHz, $CDCl_3$) δ 7.52 (dd, J = 7.7 Hz, 1H), 7.28 (dm, J = 7.9 Hz, 1H), 7.09 (dm, J = 7.6 Hz, 1H), 2.55 (s, 3H), 0.26 (s, 9H) ppm. The compound was in complete agreement with previously published data.[4]

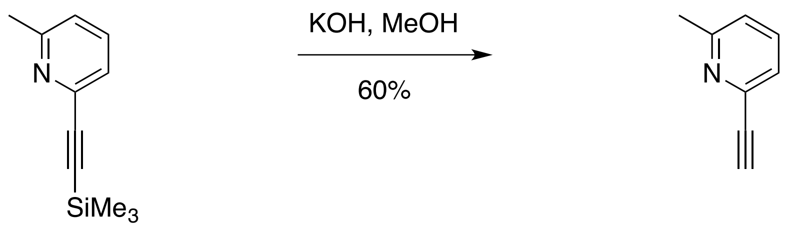

S2
$C_{11}H_{15}NSi$
(189.33)

6
C_8H_7N
(117.15)

2-Ethynyl-6-methylpyridine (6)

A yellow solution of 2-methyl-6-((trimethylsilyl)ethynyl)pyridine (226 mg, 1.19 mmol, 1eq) in methanol (0.3 mL) was treated with the solution of potassium hydroxide (134 mg, 2.39 mmol, 2 eq) in methanol (2.5 mL) and the resulting colourless solution was allowed to stir at ambient temperature over 2.5 h. After this time the crude mixture was quenched with H_2O (6 mL) and diluted with EtOAc (5 mL) and the two layers were well shaken and separated. The aqueous phase was extracted with EtOAc (3x5 mL). The combined organic extracts were washed with brine (3 mL), dried (Na_2SO_4) and concentrated *in vacuo*. The crude reaction mixture was purified by chromatography on a silica gel column (eluting with gradient 5% to 10% EtOAc/pentane) to afford the title compound (84 mg, 0.72 mmol, 60%): ^{1}H NMR (400 MHz, $CDCl_3$) δ 7.54 (dd, J = 7.8 Hz, 1H), 7.29 (dm, J = 7.8 Hz, 1H), 7.13 (dm, J = 7.8 Hz, 1H), 3.12 (s, 1H), 2.55 (s, 3H) ppm. The compound was in complete agreement with previously published data.[4]

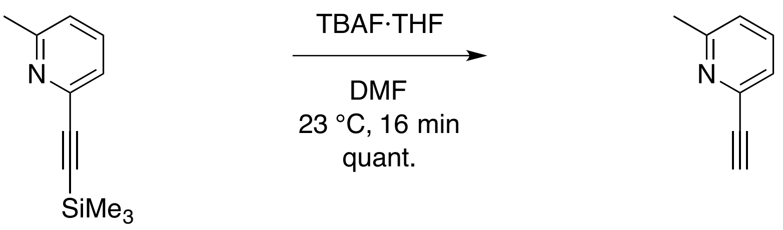

S2 $C_{11}H_{15}NSi$ (189.33)

6 C_8H_7N (117.15)

2-Ethynyl-6-methylpyridine (6)

One neck round bottom flask was charged with 2-methyl-6-((trimethylsilyl)ethynyl)pyridine (100 mg, 0.53 mmol, 1 eq), and anhydrous *N*,*N′*-dimethylformamide (2 mL) was added and the clear homogeneous solution was further treated with tetrabutylammonium fluoride solution in tetrahydrofuran (1.0 mL, 1.05 mmol, 2 eq, c=1 M) dropwise (<1 min) and the resulting dark brown mixture was allowed to stir at ambient temperature under nitrogen atmosphere over 16 min. This material without work-up or purification was used for the next step.

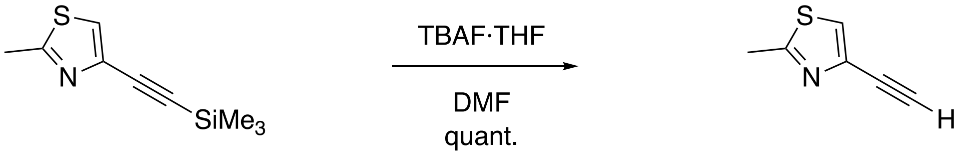

20 $C_9H_{13}NSSi$ (195.36)

10 C_6H_5NS (123.17)

4-Ethynyl-2-methylthiazole (10)

One neck round bottom flask was charged with 2-methyl-4-((trimethylsilyl)ethynyl)thiazole (103 mg, 0.53 mmol, 1 eq), and anhydrous *N*,*N′*-dimethylformamide (2 mL) was added and the clear brown homogeneous solution was further treated with tetrabutylammonium fluoride solution in tetrahydrofuran (1.0 mL, 1.06 mmol, 2 eq, c=1 M) dropwise (<1 min) and the resulting dark brown mixture was allowed to stir at ambient temperature under nitrogen atmosphere over 14 min. This material without work-up or purification was used for the next step.

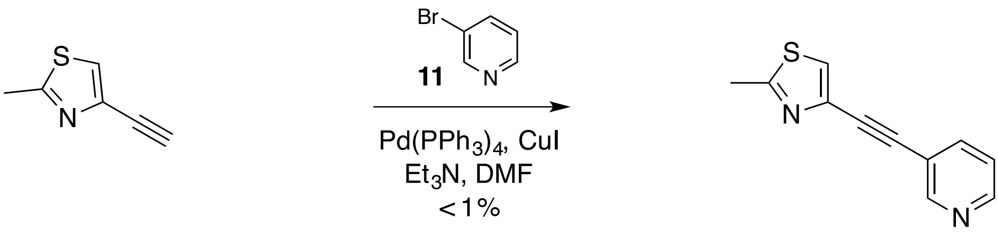

10
C_6H_5NS
(123.17)

3, MTEP
$C_{11}H_8N_2S$
(200.26)

2-Methyl-4-(pyridin-3-ylethynyl)thiazole (3)

Reaction done with the in situ formed alkyne

Two neck flask was evacuated and backfilled with nitrogen atmosphere and this was repeated three more times. This flask was charged with anhydrous *N,N′*-dimethylformamide (3.5 mL) and 3-bromopyridine (51 μL, 84 mg, 0.53 mmol, 1 eq, d=1.64) was added followed by tetrakis(triphenylphosphine)palladium(0) (18 mg, 0.016 mmol, 0.03 eq) in one portion and the bright yellow solution was allowed to stir for 10 min. After this time triethylamine (0.22 mL, 161 mg, 1.59 mmol, 3 eq, d=0.726) was added in one portion and the mixture further stirred over 12 min. After this time copper(I)iodide (10 mg, 0.053 mmol, 0.1 eq) was added in one portion and the purple mixture was further treated with a solution of crude mixture of 4-ethynyl-2-methylthiazole (65 mg, 0.53 mmol, 1 eq) in *N,N′*-dimethylformamide (2 mL) and the resulting clear brown mixture was allowed to stir at ambient temperature over 23 h. After this time the crude mixture was quenched with saturated aq. NH_4Cl (15 mL) and the mixture was diluted with H_2O (10 mL) and EtOAc (30 mL) and the two layers were well shaken and separated. The aqueous phase was extracted with EtOAc (3x30 mL). The combined organic extracts were washed with H_2O (3x25 mL), brine (30 mL), dried ($MgSO_4$) and concentrated *in vacuo* to afford crude mixture as brown oily residue. ^1H NMR and LRMS showed trace amounts of product. Due to small amount material was not further purified.

Reaction done with alkyne as isolated material

Two neck flask was evacuated and backfilled with nitrogen atmosphere and this was repeated three more times. This flask was charged with 3-bromopyridine (78 μL, 128 mg, 0.81 mmol, 1 eq, d=1.64) and anhydrous *N,N′*-dimethylformamide (1 mL) and was added followed by tetrakis(triphenylphosphine)palladium(0) (28 mg, 0.02 mmol, 0.03 eq) in one portion and the bright yellow solution was allowed to stir for 12 min. After this time triethylamine (0.34 mL, 245 mg, 2.43 mmol, 3 eq, d=0.726) was added in one portion and the mixture further stirred over 17 min. After this time copper(I)iodide (15 mg, 0.08 mmol, 0.1 eq) was added in one portion and the mixture was further treated with a solution of 4-ethynyl-2-methylthiazole (100 mg, 0.81 mmol, 1 eq) in *N,N′*-dimethylformamide (1 mL) and the resulting red/brown mixture was allowed to stir at ambient temperature over 23 h. After this time the crude

mixture was quenched with saturated aq. NH_4Cl (10 mL) and the mixture was diluted with H_2O (10 mL) and EtOAc (25 mL) and the two layers were well shaken and separated. The aqueous phase was extracted with EtOAc (2x25 mL). The combined organic extracts were washed with H_2O (3x20 mL), brine (20 mL), dried (Na_2SO_4) and concentrated *in vacuo* to afford crude mixture as brown oily residue. ^1H NMR and LRMS showed trace amounts of product. Due to small amount material was not further purified.

References

(1) Khan, Z. A.; Wirth, T. Synthesis of Indene Derivatives via Electrophilic Cyclization. *Org. Lett.* **2009**, *11* (1), 229–231.

(2) Ding, Y.; Green, J. R. Benzocycloheptynedicobalt Complexes by Intramolecular Nicholas Reactions. *Synlett* **2005**, *2005* (2), 271–274.

(3) Ding, C.; Babu, G.; Orita, A.; Hirate, T.; Otera, J. Synthesis and Photoluminescence Studies of Siloles with Arylene Ethynylene Strands. *Synlett* **2007**, *2007* (16), 2559–2563.

(4) Alagille, D.; Baldwin, R. M.; Roth, B. L.; Wroblewski, J. T.; Grajkowska, E.; Tamagnan, G. D. Synthesis and Receptor Assay of Aromatic–ethynyl–aromatic Derivatives with Potent mGluR5 Antagonist Activity. *Bioorg. Med. Chem.* **2005**, *13* (1), 197–209.

(5) Karama, U.; Höfle, G. Synthesis of Epothilone 16,17-Alkyne Analogs by Replacement of the C13−C15(O)-Ring Segment of Natural Epothilone C. *European J. Org. Chem.* **2003**, *2003* (6), 1042–1049.

(6) Cosford, N. D. P.; Tehrani, L.; Roppe, J.; Schweiger, E.; Smith, N. D.; Anderson, J.; Bristow, L.; Brodkin, J.; Jiang, X.; McDonald, I.; Rao, S.; Washburn, M.; Varney, M. A. 3-[(2-Methyl-1,3-Thiazol-4-Yl)ethynyl]-Pyridine: A Potent and Highly Selective Metabotropic Glutamate Subtype 5 Receptor Antagonist with Anxiolytic Activity. *J. Med. Chem.* **2003**, *46* (2), 204–206.

(7) Iso, Y.; Grajkowska, E.; Wroblewski, J. T.; Davis, J.; Goeders, N. E.; Johnson, K. M.; Sanker, S.; Roth, B. L.; Tueckmantel, W.; Kozikowski, A. P. Synthesis and Structure-Activity Relationships of 3-[(2-Methyl-1,3-Thiazol-4-Yl)ethynyl]pyridine Analogues as Potent, Noncompetitive Metabotropic Glutamate Receptor Subtype 5 Antagonists; Search for Cocaine Medications. *J. Med. Chem.* **2006**, *49* (3), 1080–1100.

Supporting Information: ^{1}H NMR Spectra

Improved Syntheses of mGlu$_5$ Antagonists MMPEP and MTEP Using Sonogashira Cross-Coupling

Boshuai Mu, Linjing Mu, Roger Schibli, Simon M. Ametamey and Selena Milicevic Sephton*

^{1}H NMR spectra were recorded in Fourier transform mode at the field strength specified using standard 5 mm diameter tubes. Chemical shifts in ppm is quoted relative to residual solvent signals calibrated as follows: $CDCl_3$ δ_H ($CHCl_3$) = 7.26 ppm. Spectra were collected at ambient temperature.

Compound	^{1}H NMR	page
**S1**	400 MHz, $CDCl_3$	S19
**5A**	400 MHz, $CDCl_3$	S20
**2**, MMPEP	400 MHz, $CDCl_3$	S21
**2·HCl**	400 MHz, $CDCl_3$	S22
**8**	400 MHz, $CDCl_3$	S23
**17**	400 MHz, $CDCl_3$	S24
**19**	400 MHz, $CDCl_3$	S25

Compound	^{1}H NMR	page
**20**	400 MHz, $CDCl_3$	S26
**10**	400 MHz, $CDCl_3$	S27
**3**, MTEP	400 MHz, $CDCl_3$	S28
**3·HCl**	400 MHz, $CDCl_3$	S29
**S2**	400 MHz, $CDCl_3$	S30
**6**	400 MHz, $CDCl_3$	S31

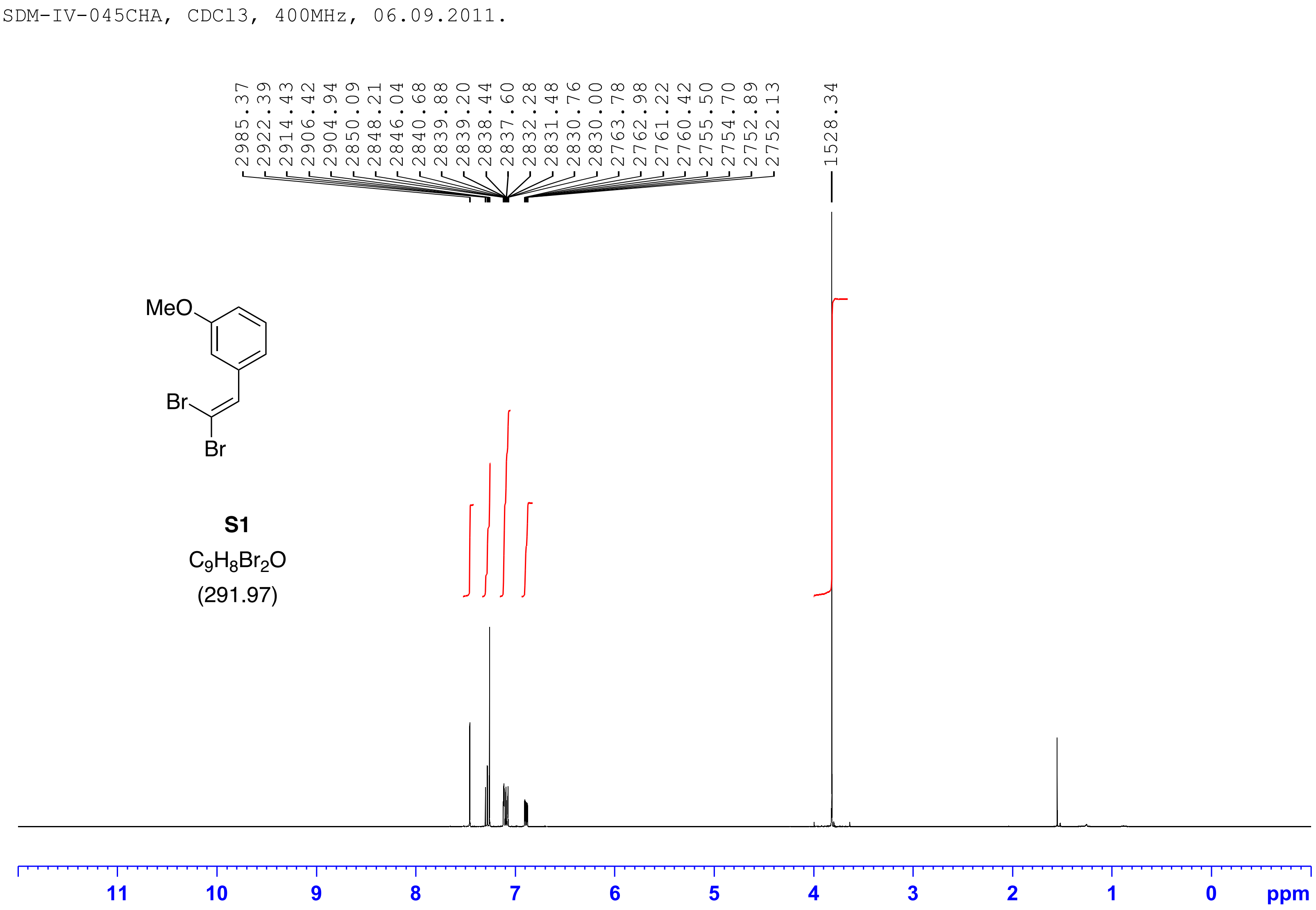
SDM-IV-045CHA, CDCl3, 400MHz, 06.09.2011.
2985.37
2922.39
2914.43
2906.42
2904.94
2850.09
2848.21
2846.04
2840.68
2839.88
2839.20
2838.44
2837.60
2832.28
2831.48
2830.76
2830.00
2763.78
2762.98
2761.22
2760.42
2755.50
2754.70
2752.89
2752.13
1528.34
MeO
Br
Br
S1
$C_9H_8Br_2O$
(291.97)
11
10
9
8
7
6
5
4
3
2
1
0
ppm

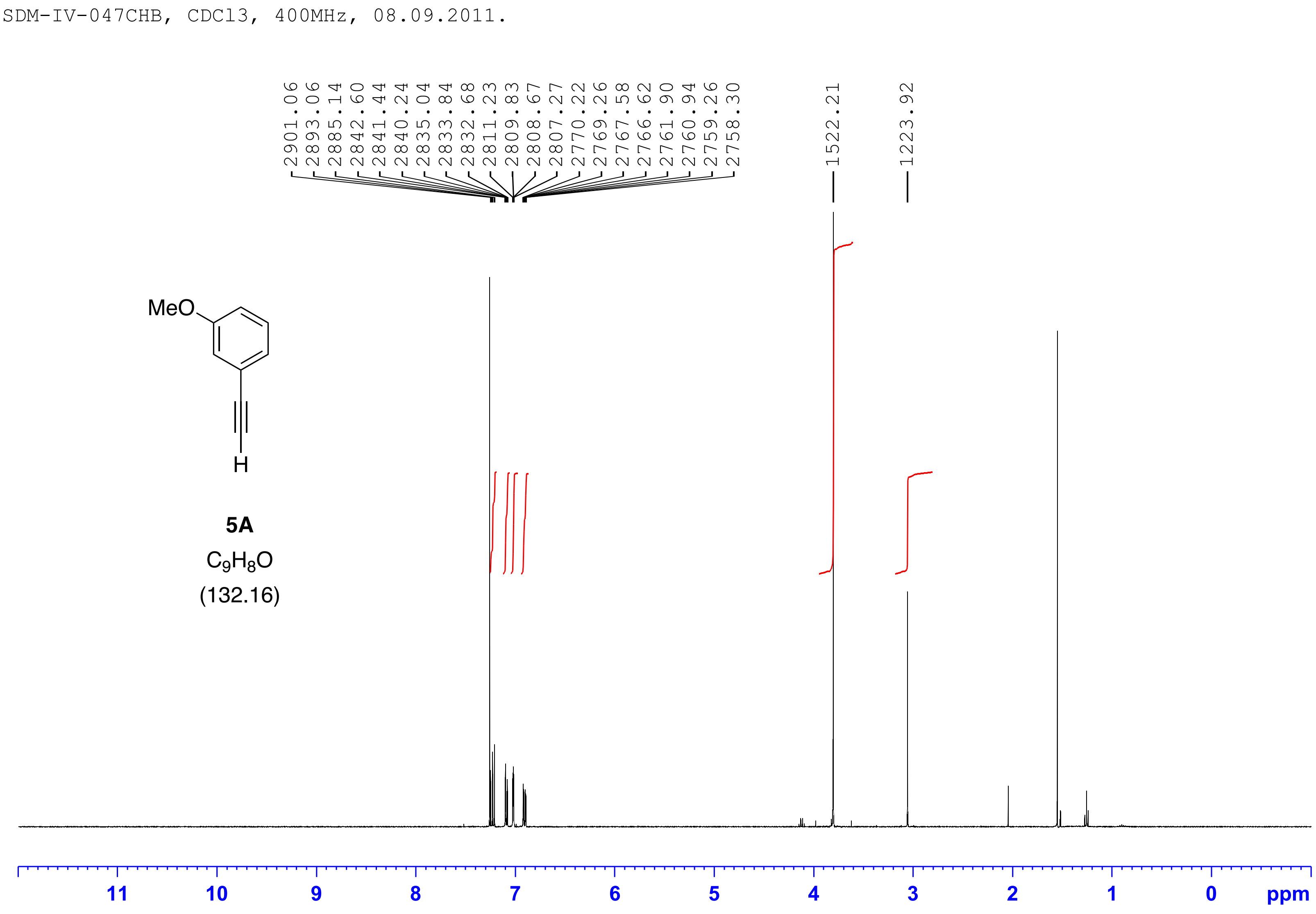
SDM-IV-047CHB, CDCl3, 400MHz, 08.09.2011.
2901.06
2893.06
2885.14
2842.60
2841.44
2840.24
2835.04
2833.84
2832.68
2811.23
2809.83
2808.67
2807.27
2770.22
2769.26
2767.58
2766.62
2761.90
2760.94
2759.26
2758.30
1522.21
1223.92
MeO
H
5A
C_9H_8O
(132.16)
11
10
9
8
7
6
5
4
3
2
1
0
ppm

SDM-IV-049CHA, CDCl3, 400MHz, 13.09.2011.

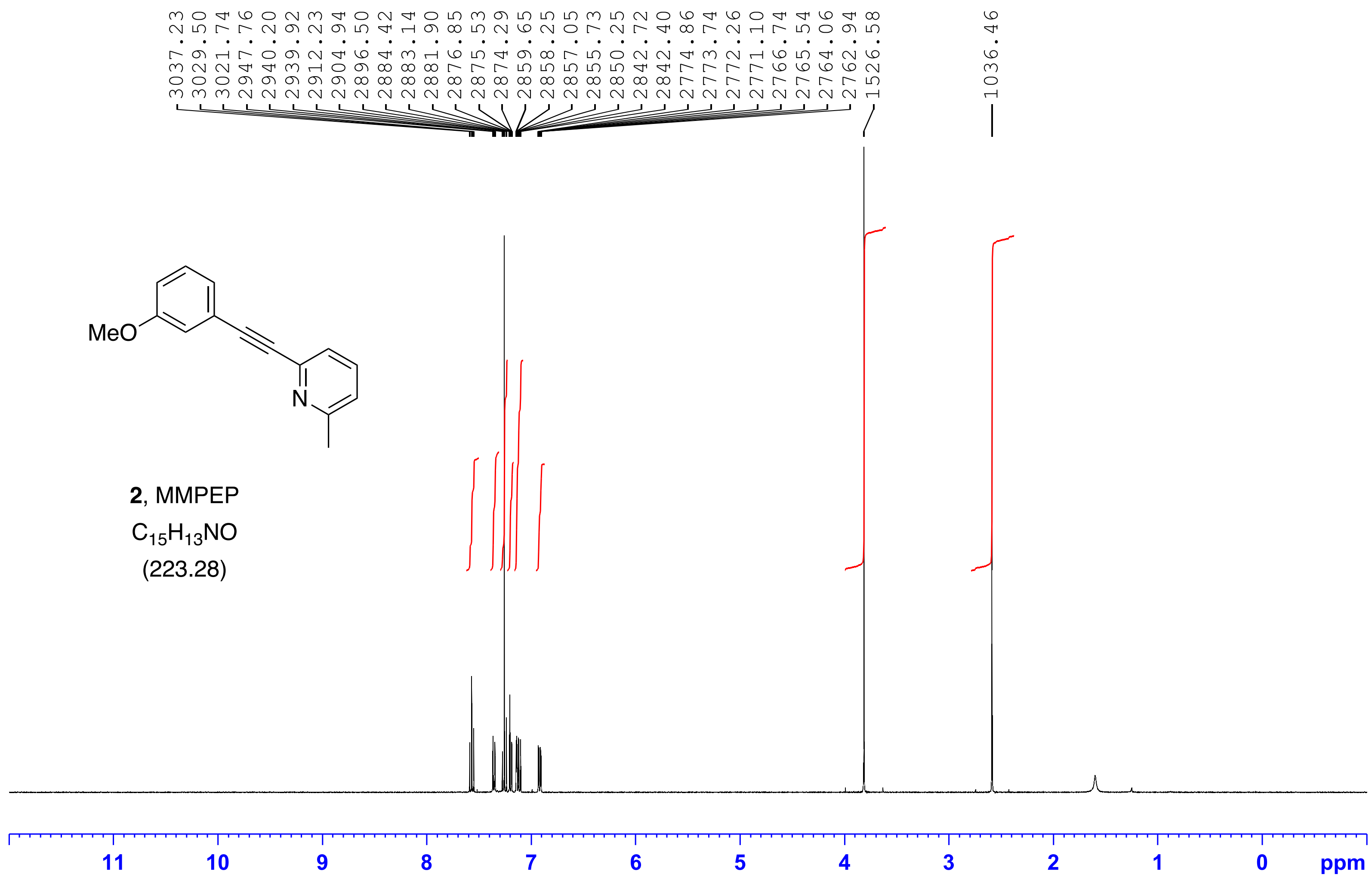

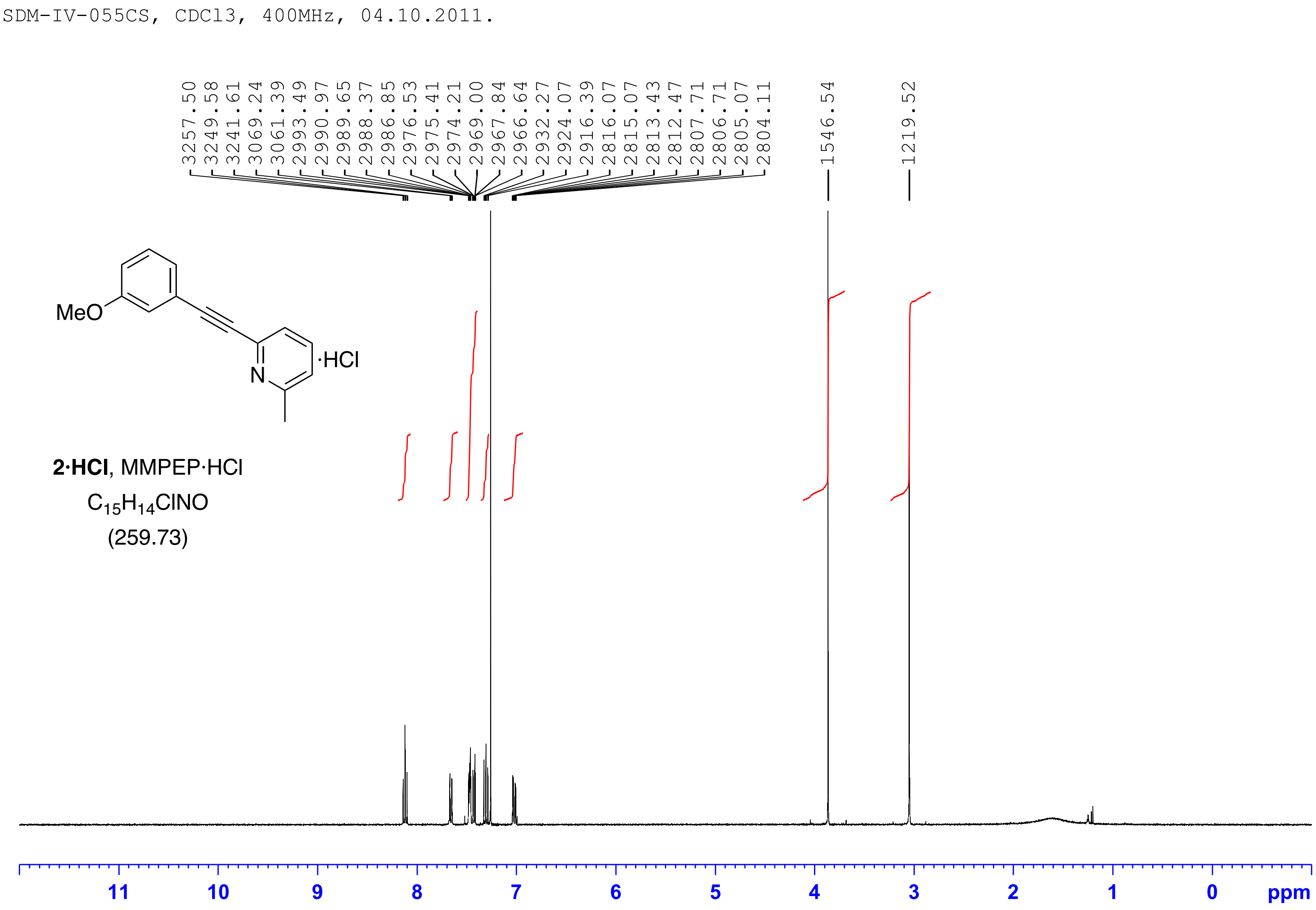

2·HCl, MMPEP·HCl

$C_{15}H_{14}ClNO$

(259.73)

SDM-III-096CHA, CDCl3, 400MHz, 23.03.2011.

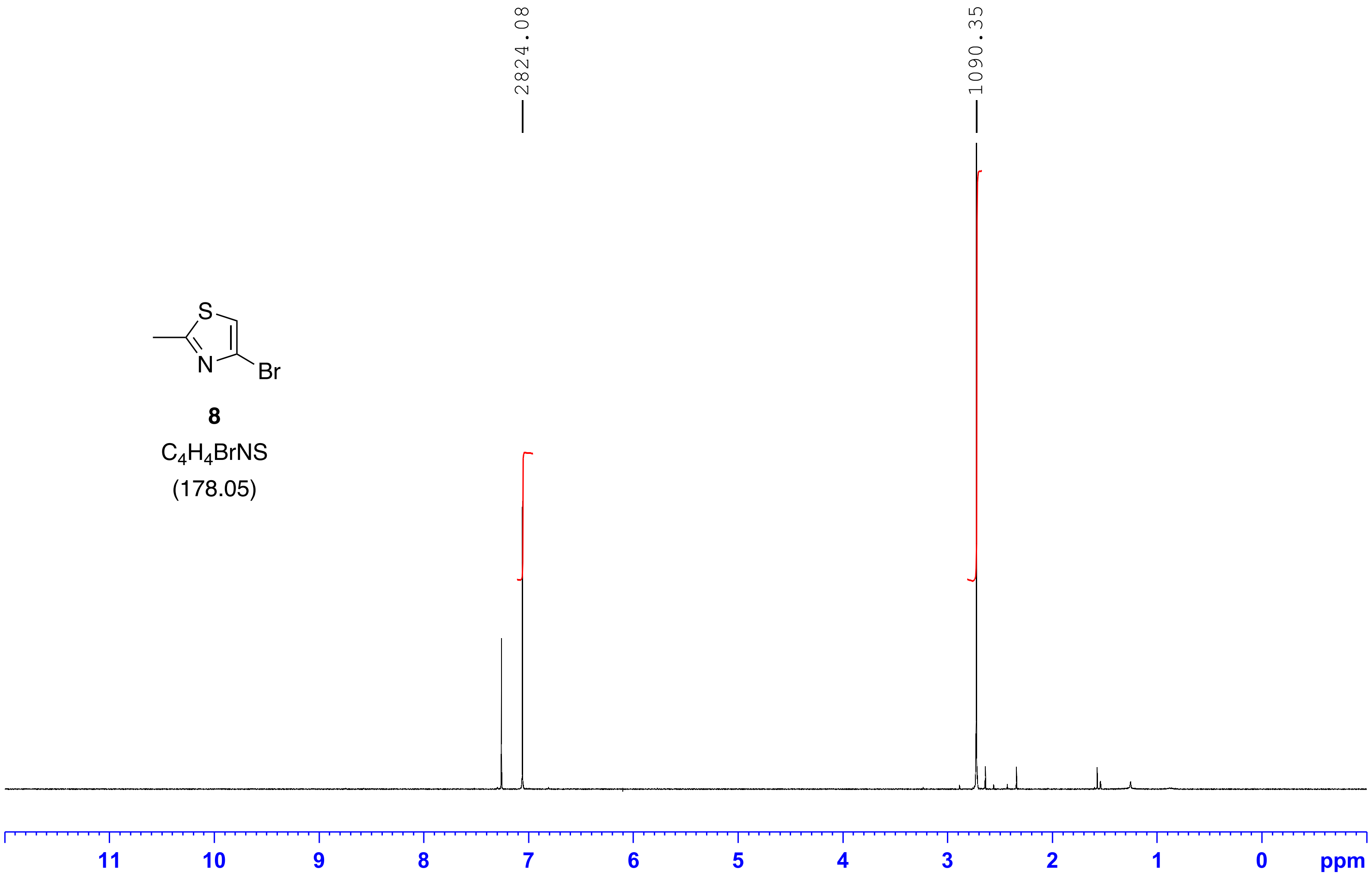

8

C_4H_4BrNS

(178.05)

SDM_V_088CHA, CDCl3, 400MHz, 31.07.2013.

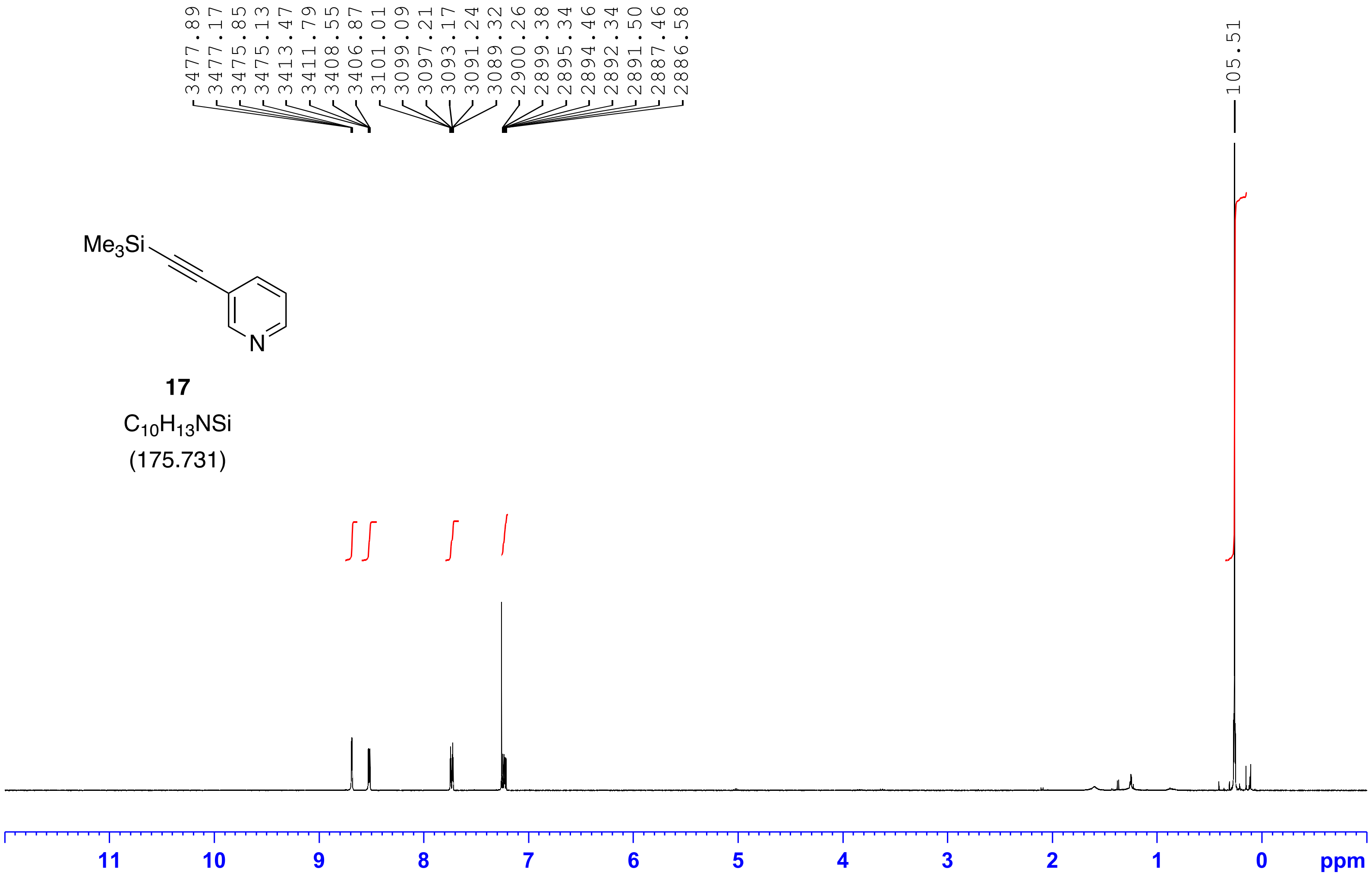

17

$C_{10}H_{13}NSi$

(175.731)

SDM-IV-080D, CDCl3, 400MHz, 10.12.2011.

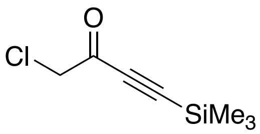

19

$C_7H_{11}ClOSi$

(174.70)

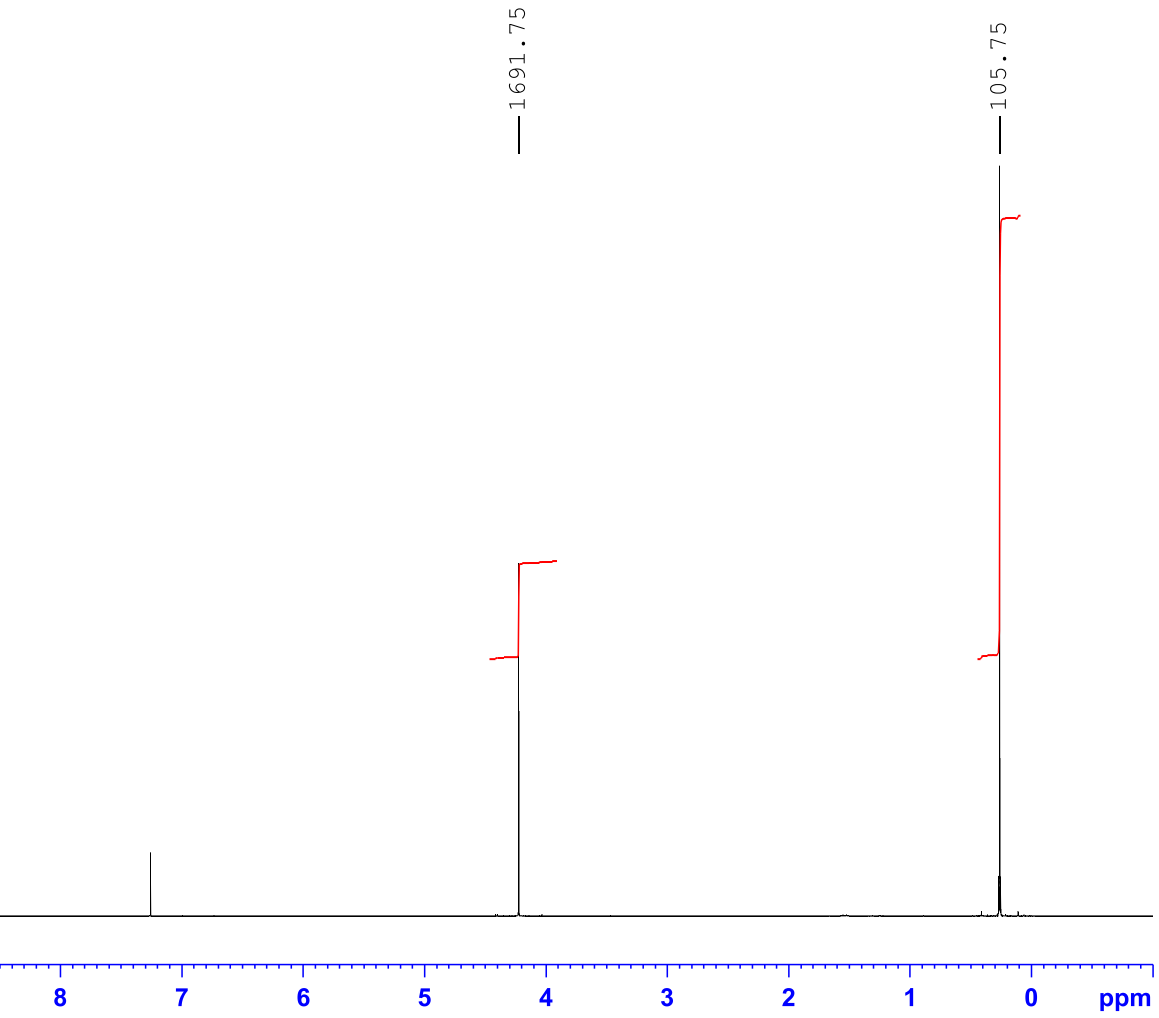

SDM-IV-082CHB, CDCl3, 400MHz, 11.12.2011.

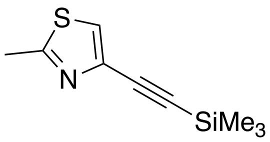

20

$C_9H_{13}NSSi$

(195.36)

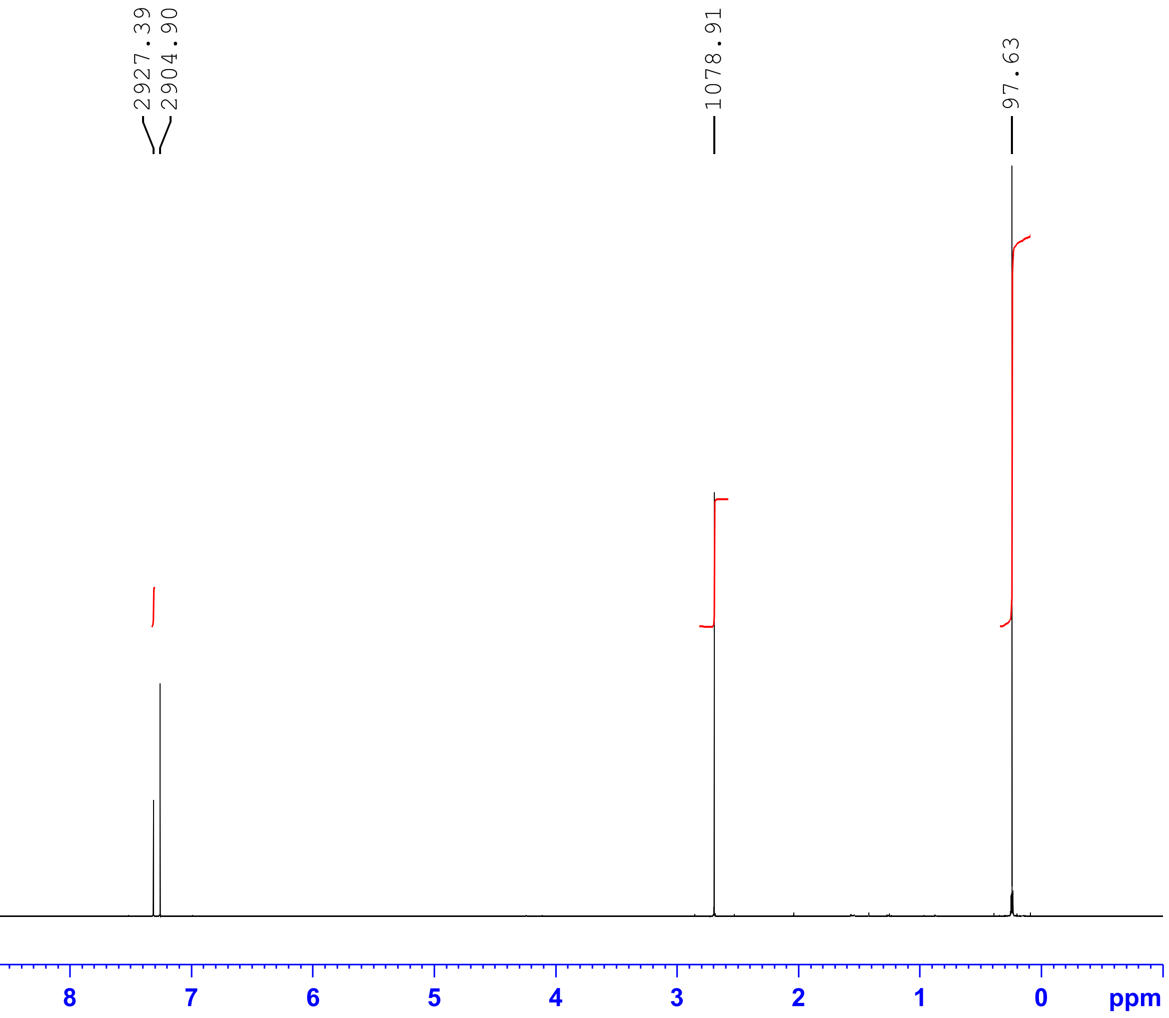

SDM_V_087CHA, CDCl3, 400MHz, 31.07.2013.

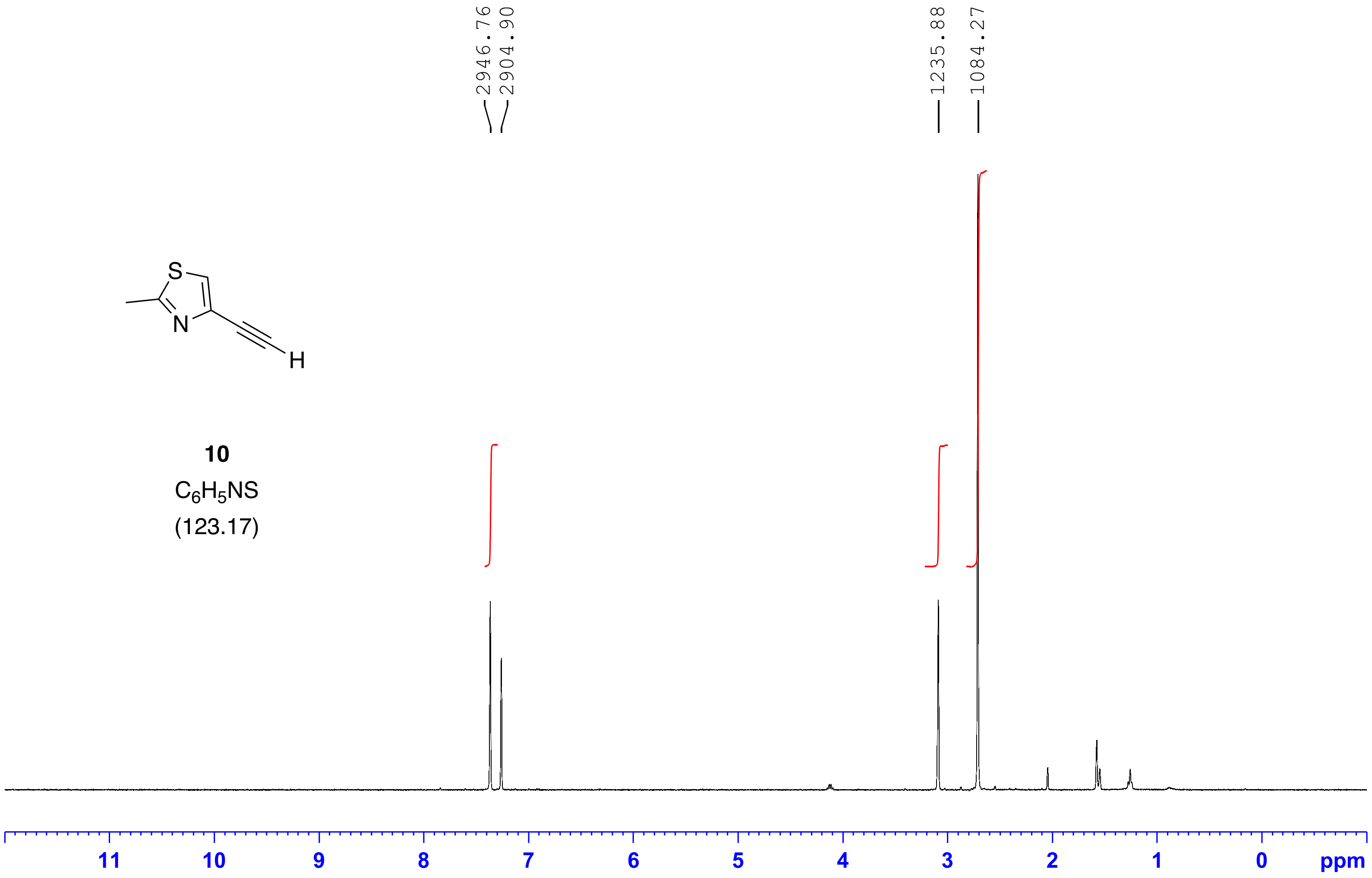

SDM-IV-083CS1, CDCl3, 400MHz, 14.12.2011.

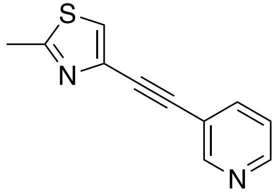

3, MTEP

$C_{11}H_8N_2S$

(200.26)

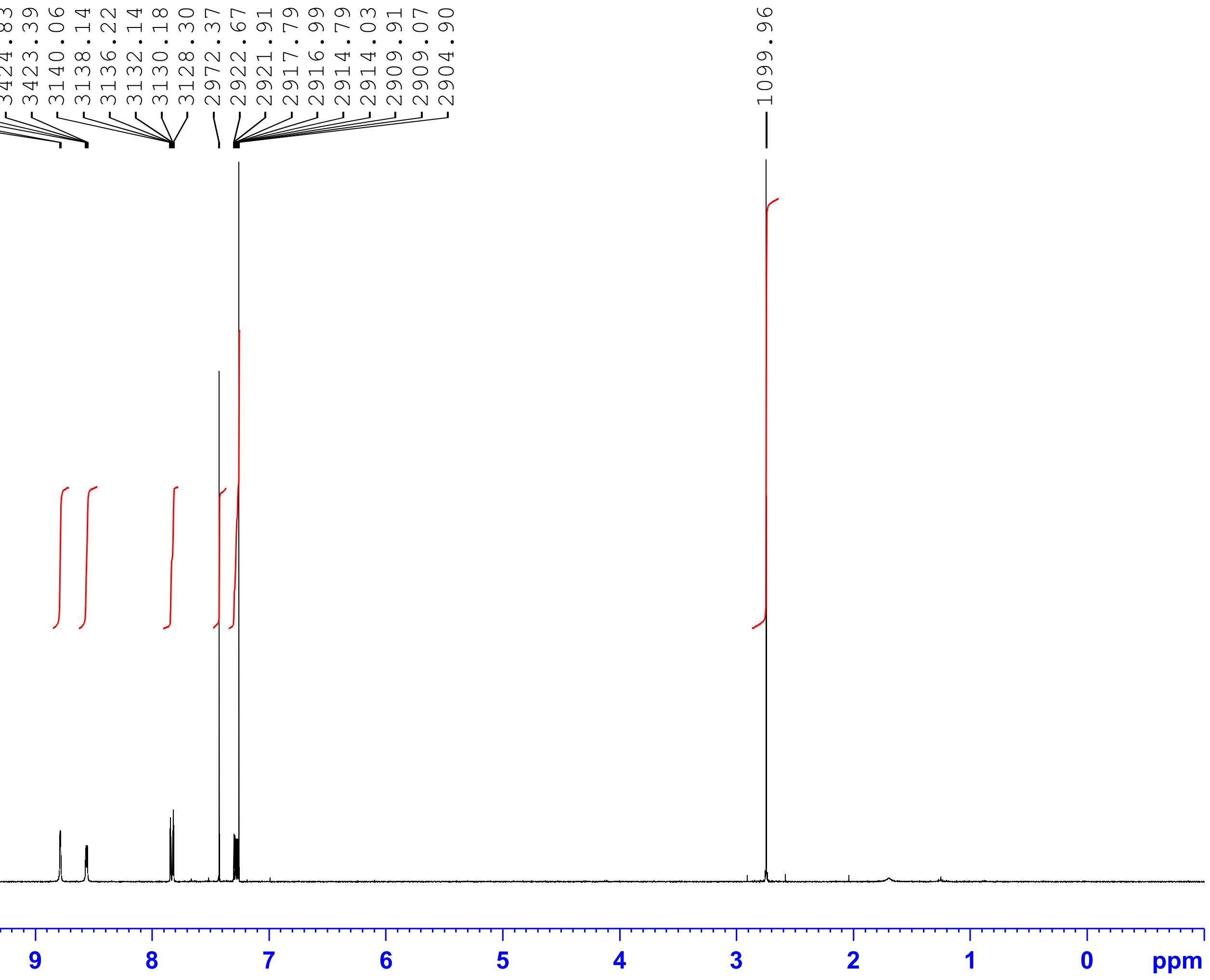

SDM-IV-086CS, CDCl3, 400MHz, 14.12.2011.

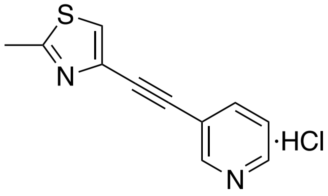

3·HCl, MTEP·HCl

$C_{11}H_9ClN_2S$

(236.72)

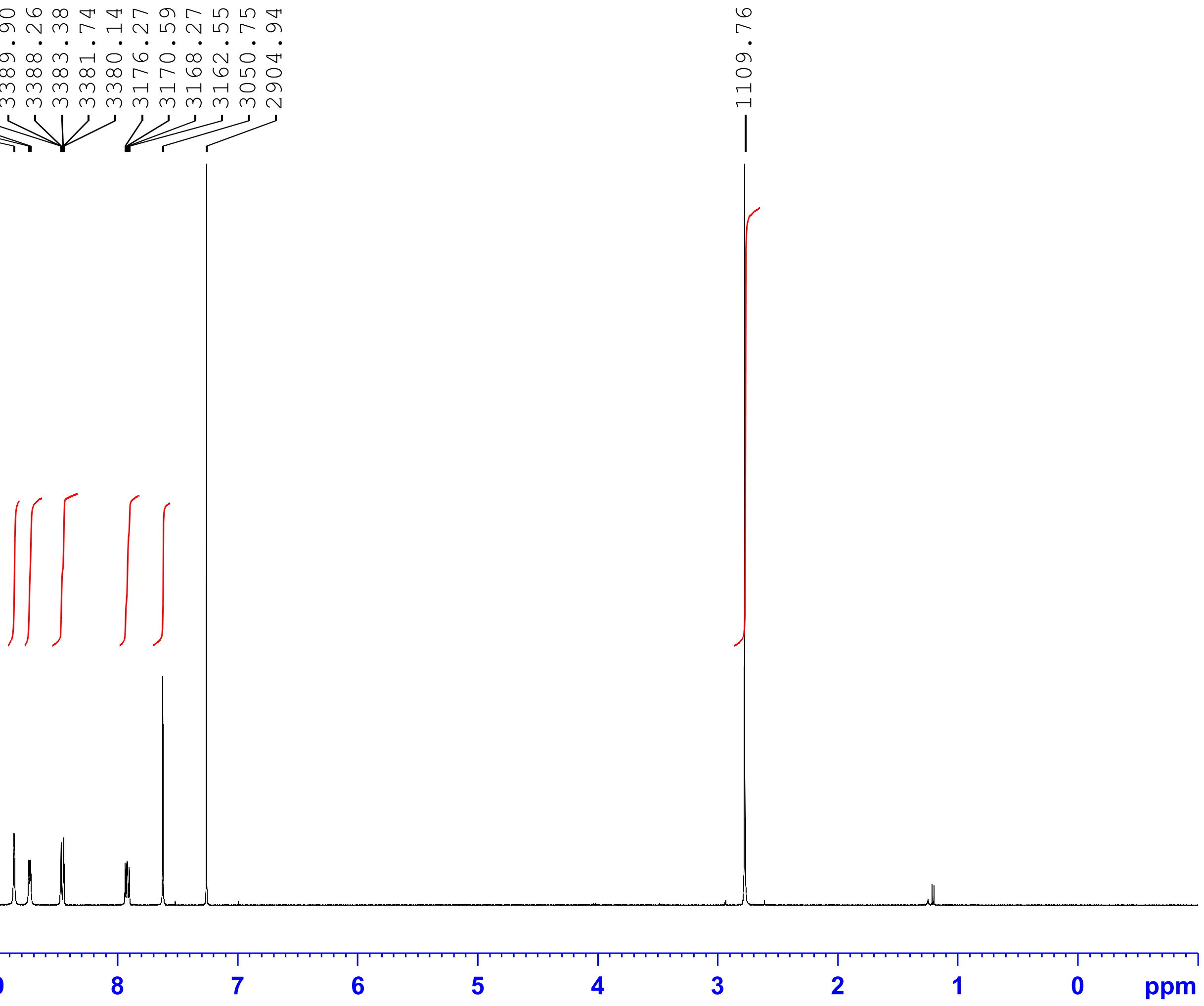

BM-I-016CH FLASK B CDCl3 400M 31-07-2013

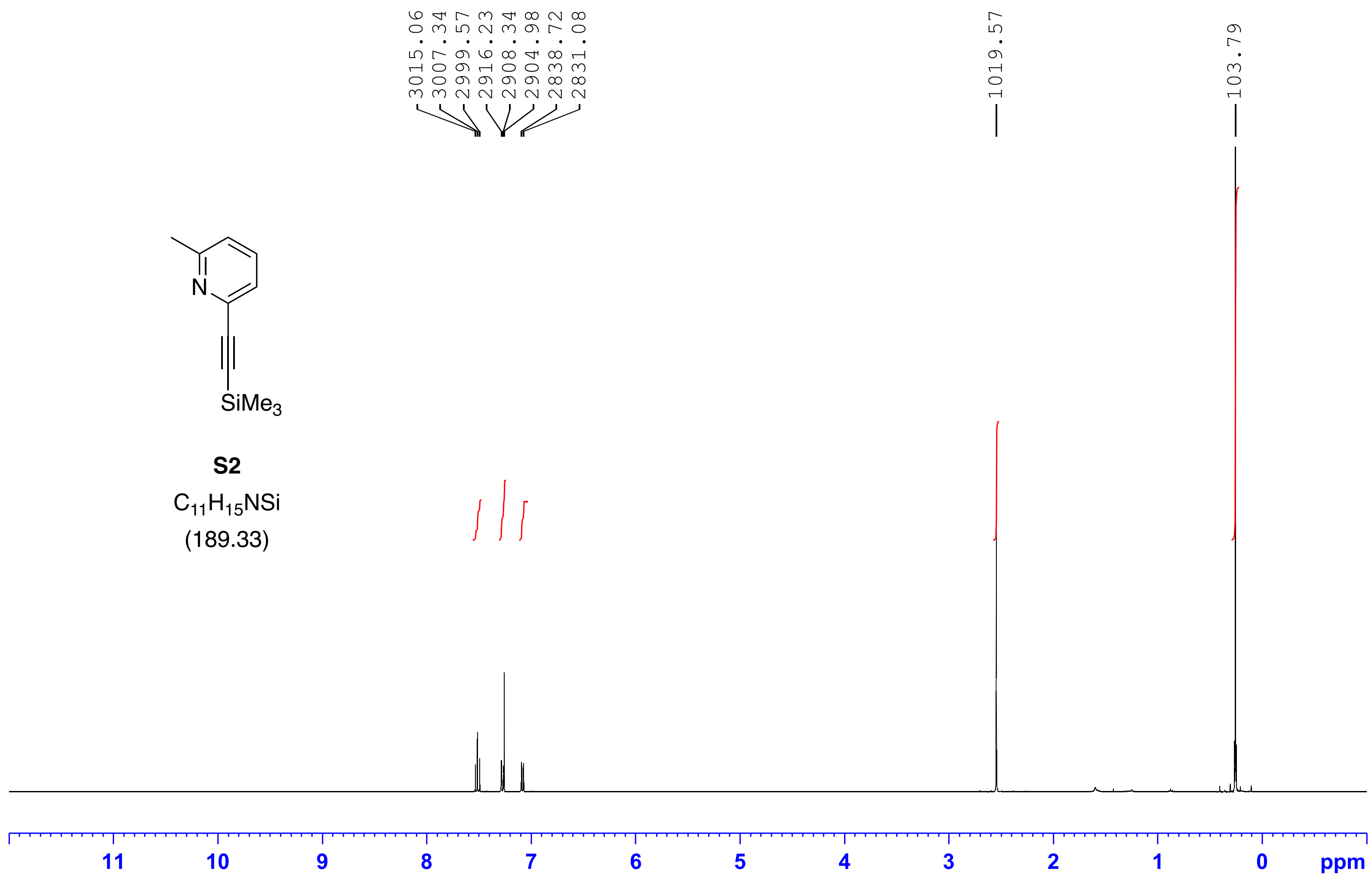

S2

$C_{11}H_{15}NSi$

(189.33)

BM-I-021CH CDCl3 400M 06-08-2013

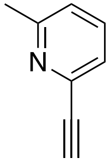

6
C_8H_7N
(117.15)

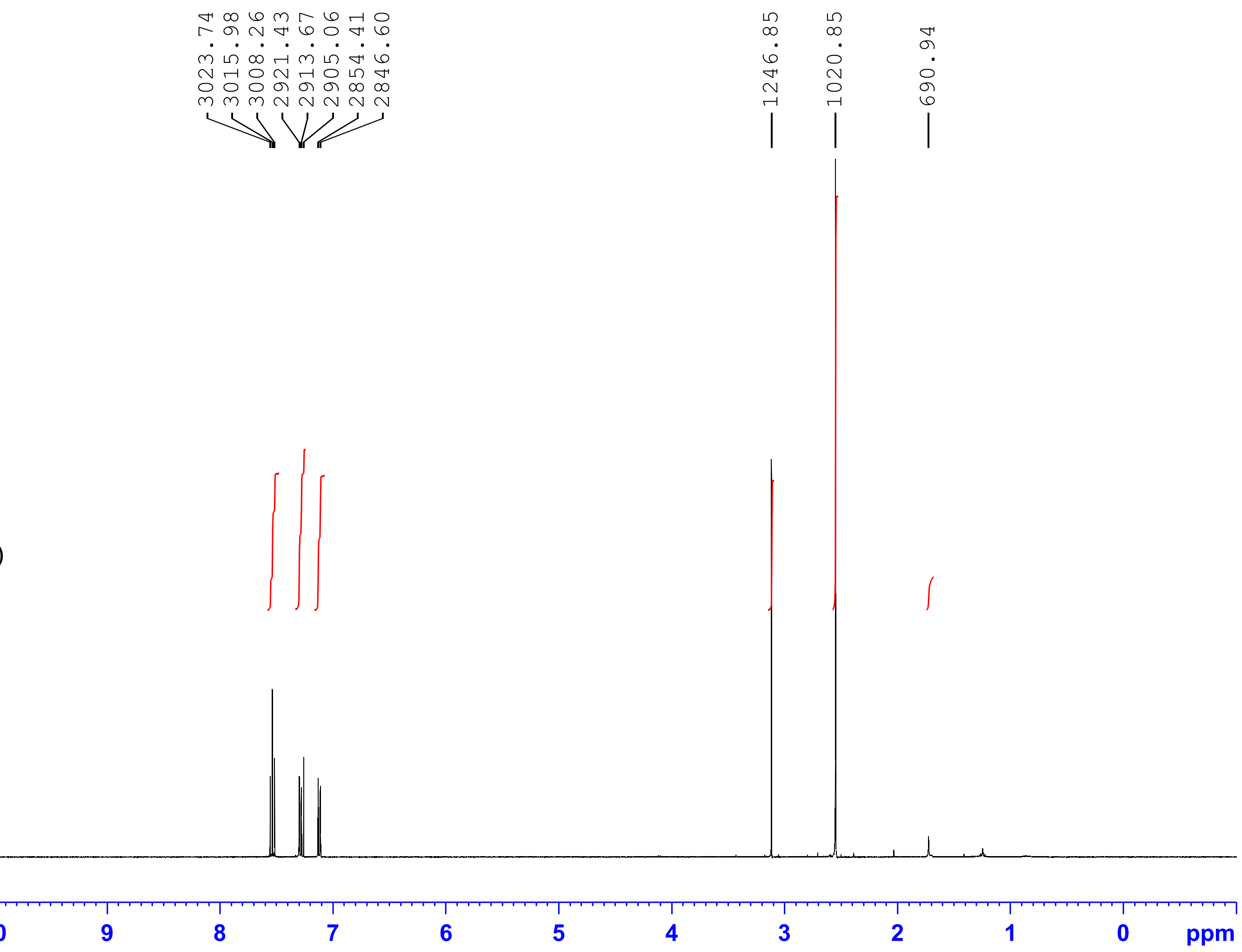

Supporting Information: Computational Data

Scope of Sonogashira Cross-Coupling in the Syntheses of $mGlu_5$ Antagonists MMPEP and MTEP

Boshuai Mu, Linjing Mu, Roger Schibli, Simon M. Ametamey and Selena Milicevic Sephton*

All calculations were performed using Density Functional Theory (DFT) from the chemistry program Spartan´14 version 1.1, Wavefunction, Inc., Irvine, CA. Optimisation of structures was performed using B3LYP method and the 6-311++G(2DF, 2P) basis set and the use of molecular symmetry was disabled. HOMO and LUMO energy calculations and electronic potential maps were obtained and Cartesian coordinates of all structures are provided below.

Compound	page
**4**	S33
**21**	S34
**11**	S35
**7A**	S36
**8**	S37
**5A**	S38

Compound	page
**6**	S39
**9**	S40
**10**	S41
**15**	S42
**22**	S43

Table S1. Data and Cartesian co-ordinates (Å) for computed ground state of **4**, GS (DFT)

HOMO

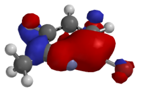

$E = -17.95 \cdot 10^5$ kcalmol^{-1}

EPM

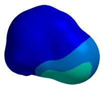

$E_{HOMO} = -7.03$ eV

$E_{LUMO} = -1.27$ eV

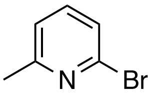

4

point group = C_S

Atom	X	Y	Z
H1	–1.8005580	–2.9084962	0.0000000
C1	–1.4887097	–1.8723108	0.0000000
N1	–0.6935035	0.7748375	0.0000000
C2	–0.1375418	–1.5545515	0.0000000
C6	–2.4311948	–0.8544818	0.0000000
C5	–1.9997689	0.4717103	0.0000000
C3	0.1819949	–0.2032852	0.0000000
H2	0.6298099	–2.3129427	0.0000000
H6	–3.4887741	–1.0804975	0.0000000
C4	–2.9699334	1.6197954	0.0000000
H4	–3.6160671	1.5858407	–0.8789308
H5	–2.4293136	2.5622798	0.0000000
H7	–3.6160671	1.5858407	0.8789308
Br1	2.0354630	0.3031045	0.0000000

Table S2. Data and Cartesian co-ordinates (Å) for computed ground state of **21**, GS (DFT)

HOMO

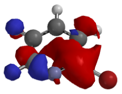

$E = -17.71 \cdot 10^5$ kcalmol^{-1}

EPM

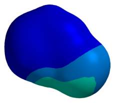

$E_{HOMO} = -7.24$ eV
$E_{LUMO} = -1.47$ eV

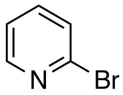

22

point group = C_S

Atom	X	Y	Z
H1	2.7350154	−2.1119914	0.0000000
C1	2.1782164	−1.1845019	0.0000000
N1	0.7352298	1.1869129	0.0000000
C2	0.7924962	−1.2082516	0.0000000
C6	2.8374027	0.0405589	0.0000000
C5	2.0730250	1.1972574	0.0000000
C3	0.1359402	0.0197778	0.0000000
H2	0.2383708	−2.1341270	0.0000000
H6	3.9164633	0.0982451	0.0000000
H5	2.5394845	2.1747945	0.0000000
Br1	−1.7841417	0.0290215	0.0000000

Table S3. Data and Cartesian co-ordinates (Å) for computed ground state of **11**, GS (DFT)

HOMO

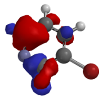

$E = -17.71 \cdot 10^5$ kcalmol^{-1}

EPM

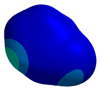

$E_{HOMO} = -7.26$ eV
$E_{LUMO} = -1.49$ eV

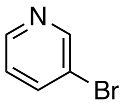

11

point group = C_S

Atom	X	Y	Z
H1	–0.2793235	–2.1539397	0.0000000
C1	–0.8064348	–1.2107856	0.0000000
N1	–2.1479741	1.2346275	0.0000000
C2	–2.1939772	–1.1592548	0.0000000
C6	–0.1151632	–0.0098235	0.0000000
C5	–0.8175289	1.1907710	0.0000000
C3	–2.8180981	0.0823303	0.0000000
H2	–2.7795757	–2.0681669	0.0000000
H5	–0.2911057	2.1370426	0.0000000
H3	–3.8989686	0.1561599	0.0000000
Br1	1.7909140	0.0088110	0.0000000

Table S4. Data and Cartesian co-ordinates (Å) for computed ground state of **7A**, GS (DFT)

HOMO

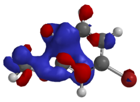

$E = -18.33 \cdot 10^5$ kcalmol^{-1}

EPM

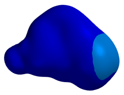

$E_{HOMO} = -6.52$ eV
$E_{LUMO} = -0.81$ eV

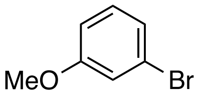

7A

point group = C_S

Atom	X	Y	Z
H1	–0.2092829	–1.7367731	0.0000000
C1	–0.3935625	–0.6732766	0.0000000
C4	–0.9064370	2.0539827	0.0000000
C2	–1.7134231	–0.2109048	0.0000000
C6	0.6402990	0.2420032	0.0000000
C5	0.4086564	1.6140180	0.0000000
C3	–1.9731142	1.1589052	0.0000000
H5	1.2312139	2.3130109	0.0000000
H3	–2.9842741	1.5350451	0.0000000
H4	–1.1093677	3.1165988	0.0000000
O1	–2.6682060	–1.1774579	0.0000000
H2	–4.0334762	–0.7906720	0.8919467
H7	–4.6046923	–0.2113732	0.0000000
H8	–4.2836669	–0.2113732	–0.8919467
Br1	2.4409083	–0.4017815	0.0000000

Table S5. Data and Cartesian co-ordinates (Å) for computed ground state of **8**, GS (DFT)

HOMO 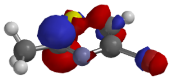 E = –19.97·10^5 kcalmol^{-1}

EPM 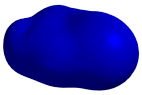 E_{HOMO} = –6.73 eV

E_{LUMO} = –1.32 eV

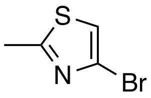 point group = C_S

8

Atom	X	Y	Z
C1	1.8415491	–0.5348636	0.0000000
S1	2.0062596	1.2019413	0.0000000
N1	0.6050826	–0.9403460	0.0000000
C2	3.0224558	–1.4488875	0.0000000
H2	2.6678914	–2.4767164	0.0000000
H4	3.6465875	–1.2930880	0.8807579
H5	3.6465875	–1.2930880	–0.8807579
C3	–0.2551400	0.1149015	0.0000000
C4	0.2928439	1.3570848	0.0000000
H7	–0.2002546	2.3120337	0.0000000
Br1	–2.1190627	–0.2144465	0.0000000

Table S6. Data and Cartesian co-ordinates (Å) for computed ground state of **5A**, GS (DFT)

HOMO

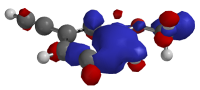

E = –26.55·10^4 kcalmol^{-1}

EPM

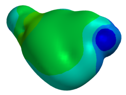

E_{HOMO} = –6.39 eV
E_{LUMO} = –1.18 eV

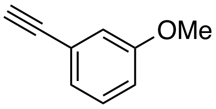

5A

point group = C_S

Atom	X	Y	Z
H1	–0.3574975	–1.8774180	0.0000000
C1	–0.2596710	–0.8017051	0.0000000
C4	0.0333773	1.9566694	0.0000000
C2	1.0166731	–0.2394670	0.0000000
C6	–1.3906702	0.0142175	0.0000000
C5	–1.2375742	1.4107822	0.0000000
C3	1.1679819	1.1467996	0.0000000
H5	–2.1129641	2.0433317	0.0000000
H3	2.1472828	1.5996453	0.0000000
H4	0.1543368	3.0316126	0.0000000
C7	–2.6930444	–0.5682511	0.0000000
H6	–4.7619218	–1.4857473	0.0000000
C8	–3.7919177	–1.0544288	0.0000000
O1	2.0489021	–1.1266297	0.0000000
C9	3.3764936	–0.6282271	0.0000000
H2	3.5785634	–0.0296710	–0.8917927
H7	4.0226982	–1.5013310	0.0000000
H8	3.5785634	–0.0296710	0.8917927

Table S7. Data and Cartesian co-ordinates (Å) for computed ground state of **6**, GS (DFT)

HOMO

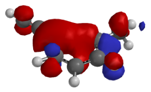

EPM

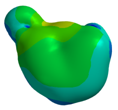

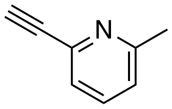

6

E = –22.83·10^{4} kcalmol^{-1}

E_{HOMO} = –6.83 eV
E_{LUMO} = –1.55 eV

point group = C_S

Atom	X	Y	Z
H1	–0.9530907	–2.8930859	0.0000000
C1	–0.7023899	–1.8405829	0.0000000
N1	–0.0698152	0.8666410	0.0000000
C2	0.6244011	–1.4363660	0.0000000
C6	–1.6990702	–0.8799360	0.0000000
C5	–1.3433078	0.4737667	0.0000000
C3	0.8974561	–0.0668448	0.0000000
H2	1.4349753	–2.1502421	0.0000000
H6	–2.7424554	–1.1660453	0.0000000
C4	2.2524722	0.3952916	0.0000000
H3	4.4062380	1.0877023	0.0000000
C7	3.3973511	0.7567511	0.0000000
C8	–2.3947314	1.5505804	0.0000000
H4	–3.0368077	1.4696555	–0.8789227
H5	–1.9223220	2.5290181	0.0000000
H7	–3.0368077	1.4696555	0.8789227

Table S8. Data and Cartesian co-ordinates (Å) for computed ground state of **9**, GS (DFT)

HOMO

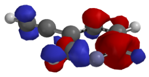

$E = –20.36·10^4$ kcalmol$^{–1}$

EPM

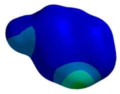

E_{HOMO} = –7.04 eV

E_{LUMO} = –1.64 eV

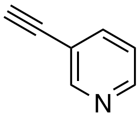

9

point group = C_S

Atom	X	Y	Z
H1	0.3679461	–2.1641032	0.0000000
C1	–0.1521298	–1.2163922	0.0000000
N1	–1.4774717	1.2376392	0.0000000
C2	–1.5355996	–1.1582240	0.0000000
C6	0.5755639	–0.0218995	0.0000000
C5	–0.1506550	1.1795574	0.0000000
C3	–2.1534938	0.0874439	0.0000000
H2	–2.1295522	–2.0615360	0.0000000
H5	0.3799905	2.1241872	0.0000000
H3	–3.2342960	0.1663858	0.0000000
C7	1.9986976	–0.0128309	0.0000000
H6	4.2618698	0.0095992	0.0000000
C8	3.2000961	–0.0013769	0.0000000

Table S9. Data and Cartesian co-ordinates (Å) for computed ground state of **10**, GS (DFT)

HOMO 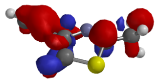 E = –42.96·10^{4} kcalmol^{-1}

EPM 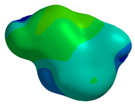 E_{HOMO} = –6.55 eV

E_{LUMO} = –1.37 eV

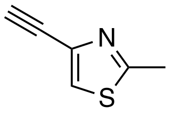

point group = C_S

10

Atom	X	Y	Z
C1	1.1667255	0.5511177	0.0000000
N1	–0.0487461	0.9972910	0.0000000
C2	–0.9734752	–0.0307790	0.0000000
C3	–0.4353929	–1.2895750	0.0000000
H5	–0.9586172	–2.2296167	0.0000000
S1	1.2744071	–1.1975301	0.0000000
C4	–2.3657735	0.2511397	0.0000000
H1	–4.5878515	0.6768616	0.0000000
C5	–3.5459344	0.4731436	0.0000000
C6	2.3831388	1.4178057	0.0000000
H2	2.0694863	2.4589187	0.0000000
H3	3.0009548	1.2376856	–0.8807088
H4	3.0009548	1.2376856	0.8807088

Table S10. Data and Cartesian co-ordinates (Å) for computed ground state of **15**, GS (DFT)

HOMO 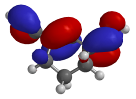 E = –27.63·10^{4} kcalmol^{-1}

EPM 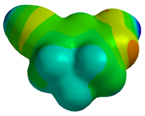 E_{HOMO} = –6.38 eV

E_{LUMO} = –1.91 eV

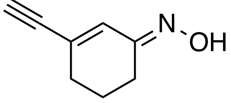

point group = C_1

15

Atom	X	Y	Z
C3	1.4572945	0.6941839	–0.0098083
C5	–0.8932438	1.5422410	0.3253112
C4	0.4191348	1.7324101	–0.4384035
C9	–1.4141014	0.1401142	0.1354908
H6	1.8480207	0.9343151	0.9850729
H10	–0.7168474	1.7081816	1.3934267
H7	0.2304954	1.6333675	–1.5102147
H5	2.3183759	0.7071718	–0.6793379
H9	–1.6528384	2.2558174	0.0111232
H8	0.8082272	2.7381107	–0.2757158
C1	0.8826295	–0.7077756	0.0289907
C2	–0.4469394	–0.9422486	0.0714276
H1	–0.8180391	–1.9553599	0.0676400
C7	1.8117132	–1.7821483	0.0202329
H2	3.3510979	–3.4433383	0.0035711
C8	2.6362726	–2.6582512	0.0112559
N1	–2.6884501	0.0033754	0.0640769
O1	–3.0873179	–1.3357284	–0.0895489
H12	–4.0454842	–1.2644386	–0.1145908

Note: During the revision process the data for compound **15** were recalculated using Spartan´16 version 2.0.9, Wavefunction, Inc., Irvine, CA. While the numerical data remain the same, the nature of HOMO orbital was different to that calculated using Spartan´14.

Table S11. Data and Cartesian co-ordinates (Å) for computed ground state of **22**, GS (DFT)

HOMO

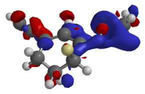

$E = -36.32 \cdot 10^4$ kcalmol^{-1}

EPM

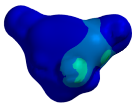

$E_{HOMO} = -6.46$ eV
$E_{LUMO} = -2.12$ eV

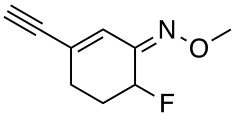

24

point group = C_1

Atom	X	Y	Z
H1	–2.9667267	0.7243992	0.9952595
C1	–2.1183094	0.8114307	0.3159482
C3	0.2233971	1.7107898	0.1091569
C9	0.7425173	0.2991301	0.0534411
C2	–1.0792553	1.7812825	0.8833829
H3	–0.8640854	1.5303416	1.9250187
H2	–2.5222763	1.1988589	–0.6235651
H5	0.9880126	2.3706728	0.5142539
H4	–1.4584914	2.8031823	0.8662696
C5	–0.2161154	–0.7825441	–0.0506448
H10	0.1597044	–1.7793345	–0.2214261
C6	–1.5452715	–0.5646876	0.0537779
C7	–2.4727154	–1.6288597	–0.0967775
H9	–4.0098239	–3.2753347	–0.3324700
C8	–3.2951402	–2.4983079	–0.2189323
N1	2.0230865	0.1857211	0.1002299
O1	2.4569296	–1.1264418	0.0388992
C4	3.8812831	–1.1591944	0.0701227
H7	4.2982459	–0.6261745	–0.7846541
H8	4.1502625	–2.2114870	0.0208064
H11	4.2564499	–0.7208160	0.9954012
F1	–0.0273775	2.1484239	–1.2095855
